# A clinical review of HIV integrase strand transfer inhibitors (INSTIs) for the prevention and treatment of HIV-1 infection

**DOI:** 10.1186/s12977-022-00608-1

**Published:** 2022-10-22

**Authors:** Alexa Vyain Zhao, Rustin D. Crutchley, Rakesh Chowdary Guduru, Kathy Ton, Tammie Lam, Amy Cheng Min

**Affiliations:** 1 Department of Pharmacy Practice and Translational Research, University of Houston, 4849 Calhoun Road Rm 3044, Houston, TX 77204-5039 USA; 2 Department of Pharmacotherapy, Washington State University College of Pharmacy and Pharmaceutical Sciences, 3110 Inspiration Dr, Yakima, WA 98901 USA; 3 GSK Medical Information Center of Excellence, 410 Blackwell Street, Durham, NC 27701 USA; 4 Department of Pharmacy Services, UC Davis Health, 2315 Stockton Blvd, Sacramento, CA 95817 USA; 5 PharmD Candidate, University of Houston College of Pharmacy, 4849 Calhoun Rd Rm 3044, Houston, TX 77204-5039 USA; 6 Department of Pharmacy Practice, Temple University School of Pharmacy, 3401 N. Broad St, Philadelphia, PA 19140 USA

**Keywords:** Bictegravir, Cabotegravir, Dolutegravir, Elvitegravir, HIV, Integrase strand transfer inhibitor (INSTI), Pre-exposure prophylaxis, Raltegravir, Treatment naïve

## Abstract

Integrase strand transfer inhibitors (INSTIs) have improved the treatment of human immunodeficiency virus (HIV). There are currently four approved for use in treatment-naïve individuals living with HIV; these include first generation raltegravir, elvitegravir, and second generation dolutegravir and bictegravir. The most recent INSTI, cabotegravir, is approved for (1) treatment of HIV infection in adults to replace current antiretroviral therapy in individuals who maintain virologic suppression on a stable antiretroviral regimen without history of treatment failure and no known resistance to its components and (2) pre-exposure prophylaxis in individuals at risk of acquiring HIV-1 infection. Cabotegravir can be administered intramuscularly as a monthly or bi-monthly injection depending on the indication. This long-acting combination has been associated with treatment satisfaction in clinical studies and may be helpful for individuals who have difficulty taking daily oral medications. Worldwide, second generation INSTIs are preferred for treatment-naïve individuals. Advantages of these INSTIs include their high genetic barrier to resistance, limited drug-drug interactions, excellent rates of virologic suppression, and favorable tolerability. Few INSTI resistance-associated mutations have been reported in clinical trials involving dolutegravir, bictegravir and cabotegravir. Other advantages of specific INSTIs include their use in various populations such as infants and children, acute HIV infection, and individuals of childbearing potential. The most common adverse events observed in clinical studies involving INSTIs included diarrhea, nausea, insomnia, fatigue, and headache, with very low rates of treatment discontinuation versus comparator groups. The long-term clinical implications of weight gain associated with second generation INSTIs dolutegravir and bictegravir warrants further study. This review summarizes key clinical considerations of INSTIs in terms of clinical pharmacology, drug-drug interactions, resistance, and provides perspective on clinical decision-making. Additionally, we summarize major clinical trials evaluating the efficacy and safety of INSTIs in treatment-naïve patients living with HIV as well as individuals at risk of acquiring HIV infection.

## Background

Extraordinary progress has been made over the past two decades in the treatment of human immunodeficiency virus (HIV). The HIV population demographics have shifted to an aging population, especially in developed countries. This dynamic can be attributed to the development of newer antiretroviral (ARV) classes such as integrase strand transfer inhibitors (INSTIs) and their availability as fixed-dose, co-formulated, single tablet, once daily products. These newer agents are characterized by excellent efficacy, better tolerability, and a majority having fewer drug-drug interactions (DDIs) than non-nucleoside reverse transcriptase inhibitor (NNRTI)- or protease inhibitor (PI)-based regimens. Given these favorable properties of INSTIs, this has led to better adherence and treatment outcomes. The role of INSTIs is particularly important in an aging HIV population with increased comorbidities and likelihood of polypharmacy. In addition, INSTIs are now being recommended worldwide in most countries as a first-line treatment for people living with HIV [1].

Treatment of HIV in individuals who are newly diagnosed with HIV (treatment-naïve) usually includes two nucleoside reverse transcriptase inhibitors (NRTIs) as backbone therapy in combination with either an INSTI, NNRTI, or a boosted PI. Recommendations on which ARV regimens are preferred for treatment-naïve individuals living with HIV are included in major standard guidelines such as the Department of Health and Human Services (DHHS), British HIV Association, European AIDS Clinical Society (EACS), and World Health Organization (WHO). Most of these guidelines recommend using INSTI-based antiretroviral therapy (ART) as a preferred option for treatment of people living with HIV [1–4]. Several clinical studies have shown INSTIs to be superior to NNRTI and PI comparative treatment groups [5–8].

**Fig. 1:**
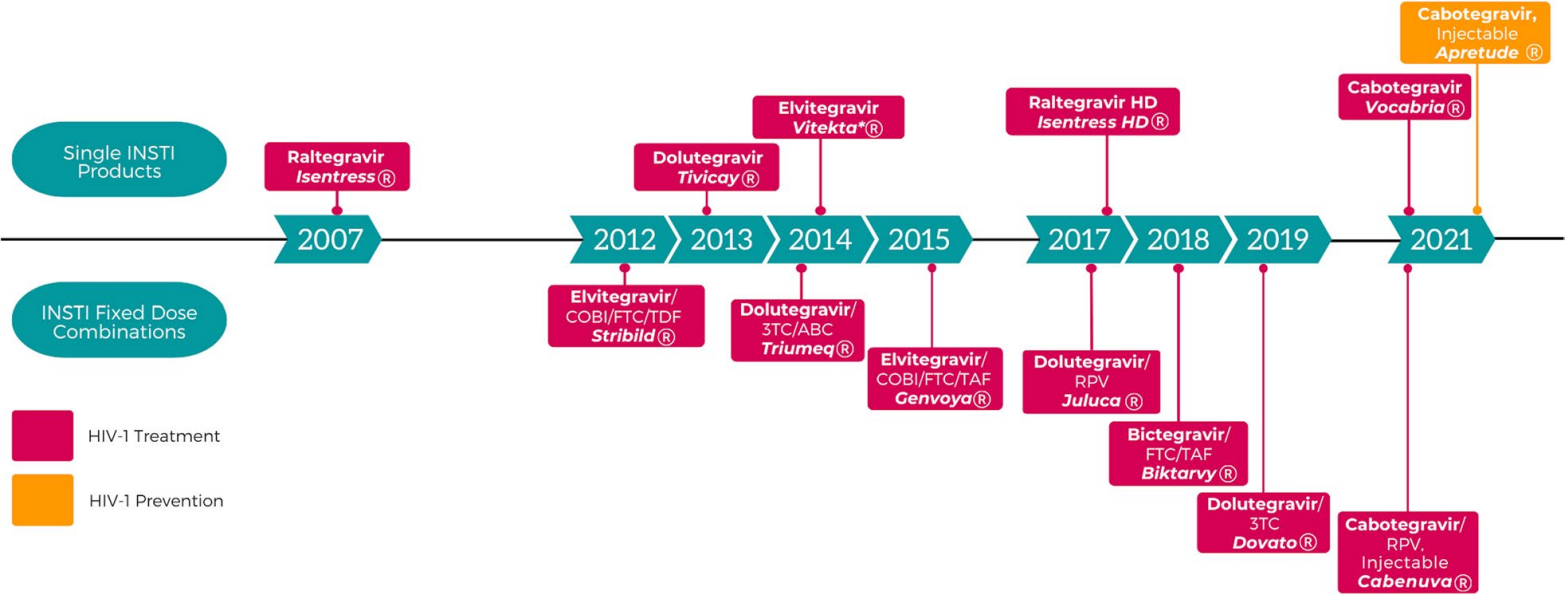
FDA approval timeline of INSTI single and fixed dose combination products used for HIV-1 treatment and prevention *discontinued, *3TC* lamivudine, *ABC* abacavir, *COBI* cobicistat, *FTC* emtricitabine, *HD* high dose, *RPV* rilpivirine, *TAF* tenofovir alafenamide, *TDF* tenofovir disoproxil fumarate

To describe the evolution of INSTIs, a timeline of the approval history of each INSTI product by the U.S. Food and Drug Administration (FDA) for both HIV-1 treatment and prevention is illustrated in Fig. 1. Raltegravir (RAL; Isentress^®^) was the first INSTI approved by the FDA for the treatment of individuals with HIV. Twice daily dosing has been problematic for some individuals, but with the approval of the 600 mg high-dose tablet (Isentress HD®) in 2017, this allowed for once daily dosing with RAL, albeit with two tablets to achieve a daily dose of 1200 mg. Other advantages of this ARV include fewer drug-drug interactions because of its metabolism primarily mediated by uridine diphosphate glucuronosyltransferase (UGT)1A1 [9], approved indication in children living with HIV as early as birth, and availability in multiple various dosage formulations. Following in 2012, elvitegravir (EVG) was approved for the treatment of individuals with HIV. EVG undergoes metabolism predominantly by cytochrome P450 (CYP) enzymes and reaches adequate concentrations ideal for once-daily dosing when combined with a potent CYP3A4 inhibitor. For this reason, this ARV is used together with cobicistat (COBI; Tybost^®^), an alternative pharmacoenhancer/booster to ritonavir (RTV; Norvir^®^), to increase its concentrations [10, 11]. Because COBI has better solubility than RTV, this has allowed for increased opportunities for co-formulation with other ARVs such as EVG 150 mg/ COBI 150 mg/tenofovir disoproxil fumarate (TDF) 300 mg/emtricitabine (FTC) 200 mg (Stribild^®^) and EVG 150 mg/COBI 150 mg/tenofovir alafenamide (TAF) 10 mg/FTC 200 mg (Genvoya^®^). Some disadvantages of EVG include potential for increased risk of DDIs, and similar to RAL, its characteristic low-genetic barrier to resistance involving a single mutation and propensity for cross-resistance with RAL [12].

Dolutegravir (DTG; Tivicay^®^) was the next approved INSTI in 2013. Advantages of DTG include its small pill size, approved indication for use as a dispersible tablet for oral suspension in infants living with HIV as young as four weeks of age and weighing at least 3 kg, high genetic barrier to resistance, and relatively few DDIs (primarily UGT1A1 metabolism). DTG is available in a single-tablet regimen including DTG 50 mg/abacavir (ABC) 600 mg/lamivudine (3TC) 300 mg (Triumeq^®^), however some limitations of this combination’s use in clinical practice include the requirement for baseline HLA-B*5701 pharmacogenetic testing to rule out risk for hypersensitivity reaction with abacavir as well as its large pill size. Recently in 2019, DTG/3TC (Dovato^®^) received FDA approval for treatment of people with HIV. The absence of abacavir in this single-tablet, one NRTI fixed dose combination (FDC) is advantageous, averting the need for HLA-B*5701 pharmacogenetic testing with ABC. The next INSTI, bictegravir (BIC), was approved by the FDA in 2018 as the co-formulated product BIC 50 mg/TAF 25 mg/FTC 200 mg (Biktarvy^®^) one tablet once daily. Advantages with this combination include small pill size, high genetic barrier to resistance, few DDIs (CYP3A and UGT1A1 mediated metabolism), dual coverage of HIV and hepatitis B virus (HBV), and its co-formulation with TAF (i.e., reduced bone and renal toxicities). Another advantage includes a low-dose FDC of BIC 30 mg/TAF 15 mg/FTC 120 mg (Biktarvy PD^®^) one tablet once daily for children weighing at least 14 kg. Disadvantages of incorporation of TAF in this FDC include an increased risk for dyslipidemia and potentially weight gain [13].

Finally, the most recent INSTI approved by the FDA in 2021 is cabotegravir (CAB). This INSTI is unique in that CAB is the first long-acting injectable suspension. Co-packaged with the NNRTI rilpivirine (RPV), it is approved as a complete regimen (Cabenuva^®^) for the treatment of HIV-1 infection in adults to replace the current ART regimen in those who are virologically suppressed (HIV-1 RNA < 50 copies/mL) on a stable ART regimen with no history of treatment failure and with no known or suspected resistance to either CAB or RPV [14]. Advantages of this two-drug combination includes its less frequent dosing of administration of intramuscular (IM) injections (i.e., once every two months or once a month), especially for individuals with problems maintaining adherence, its relatively high-genetic barrier to resistance, as well as its sparing of NRTIs. Some disadvantages include the use of an optional four week lead-in oral dosing (CAB 30-mg tablet; Vocabria^®^) together with RPV, its daily requirement for administration with a meal during the oral lead-in, the need for monthly or every two months dosing visits/injections administered by a healthcare professional, and the potential risk of resistance development in individuals unable to adhere to the administration schedule [14]. Most recently was CAB’s approval for a second indication (Apretude^®^): in individuals weighing ≥ 35 kg who are at risk of sexually acquiring HIV-1 infection. CAB can also be used with or without a lead-in with oral CAB for this indication. Refer to Table 1 for a summary of pertinent clinical characteristics of the INSTIs [14–33].

**Table 1:** Summary of Pertinent Clinical Characteristics of Available Integrase Strand Transfer Inhibitor (INSTI) Products

INSTI Product Component(s): Drug and Strength	US Trade Name	Dosage form(s) available	FDA-Labeled Indication(s)	Can it be crushed?	Metabolism	Dosing and Administration	Daily Pill Burden	Tablet Size (mm)	Reference(s)
BIC 50 mg FTC 200 mg TAF 25 mg	Biktarvy^®^	Oral FDC tablet Low dose alternative tablet: 30 mg BIC/ 120 mg FTC/ 15 mg TAF	HIV-1 Treatment: Adult and pediatric patients weighing > 25 kg with no ARV treatment history to replace current ARV regimen in those with HIV-1 RNA < 50 copies/mL on a stable ARV regimen with no history of treatment failure and no known resistance to its components	Conflicting evidence *Notes:* Potentially better to dissolve in water and take immediately rather than to crush	BIC: CYP3A UGT1A1	Adults and pediatric patients weighing at least 25 kg: 1 tablet (BIC 50 mg/FTC 200 mg/TAF 25 mg) once daily PO with or without food Pediatric patients weighing at least 14 kg and < 25 kg: 1 tablet (BIC 30 mg/FTC 120 mg/TAF 15 mg) once daily PO with or without food	1	15 × 8	[15–18]
CAB 30 mg	Vocabria^®^	Oral tablet: 30 mg	HIV-1 Treatment: Adults and adolescents 12 years of age and older weighing at least 35 kg with HIV-1 RNA < 50 copies/mL on a stable ARV regimen with no history of treatment failure and with no known or suspected resistance to CAB or RPV; indicated for short-term treatment in combination with RPV HIV-1 Prophylaxis: At-risk adults and adolescents weighing at least 35 kg for PrEP to reduce the risk of sexually acquired HIV-1 infection and who have a negative HIV-1 test prior to initiating	No *Notes:* Not recommended due to lack of evidence of efficacy and safety.	CAB: UGT1A1 UGT1A9 (minor)	HIV-1 Treatment: *Optional oral lead-in:* 1 tablet (CAB 30 mg) PO once daily in combination with 1 RPV (25 mg) tablet PO once daily with a meal for at least 28 days HIV-1 Prophylaxis: *Optional oral lead-in:* 1 tablet (CAB 30 mg) PO once daily for at least 28 days	1	14.3 × 8	[19]
CAB LA 200 mg/mL	Apretude^®^	Extended-release injectable suspension: 600 mg/3 mL	HIV-1 Prophylaxis: At-risk adults and adolescents weighing at least 35 kg for PrEP to reduce the risk of sexually acquired HIV-1 infection and who have a negative HIV-1 test prior to initiating	N/A		Initiation injection: CAB LA 3 mL once (if taking oral lead-in: on last day of oral lead-in or within 3 days thereafter) at month 2 and month 3 Continuation injections: CAB LA 3 mL at month 5 and every 2 months onwards	N/A;every 2-month injection	N/A	[19]
CAB LA 200 mg/mL plus RPV 300 mg/mL	Cabenuva^®^	Extended-release injectable suspension 400 mg/600 mg kit: CAB 400 mg/2 mL RPV 600 mg/2 mL Extended-release injectable suspension 600 mg/900 mg kit: CAB 600 mg/3 mL RPV 900 mg/3 mL	HIV-1 Treatment: Adult and pediatric patients 12 years of age and older weighing > 35 kg to replace current ARV regimen in those with HIV-1 RNA < 50 copies/mL an a stable ARV regimen with no history of treatment failure and no known resistance to its components	N/A		Dosing Schedule for Monthly Injections: *Initiation Injections:* CAB 600 mg (3 mL) IM and RPV 900 mg (3 mL) IM once given on last day of current ARV therapy OR if oral lead-in is used *Continuation injections:* CAB 400 mg (2 mL) IM and RPV 600 mg (2 mL) IM once monthly Dosing Schedule for Every-2 Month Injections: *Initiation Injections:* CAB 600 mg (3 mL) and RPV 900 mg (3 mL) IM at month 1 given on last day of current ARV therapy OR if oral lead-ins used and at month 2 *Continuation Injections:* CAB 600 mg (3 mL) and RPV 900 mg (3 mL) IM every 2 months onwards (starting at month 4)	N/A;every 2-month injection	N/A	[14]
DTG 50 mg	Tivicay^®^	Oral tablet: 10 mg 25 mg 50 mg Tivicay PD tablets for oral suspension: 5 mg	HIV-1 Treatment: Adults and pediatric patients aged at least 4 weeks and weighing at least 3 kg in combination with other antiretroviral agents	Yes *Notes:* Add to small amount of semi-solid food or liquid, consume immediately	DTG: UGT1A1 (major), CYP3A (minor)	Adults: 1 tablet (DTG 50 mg) tablet once daily PO with or without food *Take twice daily if:* INSTI resistance or also taking certain UGT1A or CYP3A inducers Pediatric: Weight based dosing (refer to prescribing information) PO with or without food *Take twice daily if:* Also taking certain UGT1A or CYP3A inducers	1 or 2	9 × 9	[20]
DTG 50 mg 3TC 300 mg	Dovato^®^	Oral FDC tablet	HIV-1 Treatment: Adults with no ARV treatment history and with no known resistance to its components	Yes *Notes:* Crushing not recommended in product information, however if crushing is decided based on clinical judgment, add to small amount of semi-solid food or liquid, consume immediately. Study data shows virologic suppression after crushing DTG when suspending it in water or adding to small amount of semi-solid food and consuming immediately		1 tablet (DTG 50 mg/3TC 300 mg) once daily PO with or without food	1	18.5 × 9.5	[21–25]
DTG 50 mg RPV 25 mg	Juluca^®^	Oral FDC tablet	HIV-1 Treatment: Adults to replace current ARV regimen in those with HIV-1 RNA ≤ 50 copies/mL on a stable ARV regimen for at least 6 months with no history of treatment failure and no known resistance to its components	No *Notes:* If crushing is decided based on clinical judgment, add to small amount of semi-solid food or liquid, consume immediately		1 tablet (DTG 50 mg/RPV 25 mg) once daily PO with a meal *Dose adjustments recommended:*Take with an additional 25-mg tablet of RPV PO once daily with a meal if coadministered with rifabutin	1	14 × 7	[26]
DTG 50 mg 3TC 300 mg ABC 600 mg	Triumeq^®^	Oral FDC tablet Triumeq PD tablets for oral suspension: DTG 5 mg/ABC 60 mg/3TC 30 mg	HIV-1 Treatment: Adult and pediatric patients weighing > 10 kg	Yes *Notes:* Add to small amount of semi-solid food or liquid, consume immediately		Adults: 1 tablet (DTG 50 mg/ABC 600 mg/3TC 300 mg) once daily PO with or without food Pediatric: Weight based dosing (refer to prescribing information) PO with or without food *DTG dose adjustments recommended:* - Also taking certain UGT1A or CYP3A inducers	1	22 × 11	[24, 25, 27]
EVG 150 mg COBI 150 mg FTC 200 mg TAF 10 mg	Genvoya^®^	Oral FDC tablet	HIV-1 Treatment: Adult and pediatric patients weighing > 25 kg with no ARV treatment history or to replace current ARV regimen in those with HIV-1 RNA < 50 copies/mL on a stable ARV regimen for at least 6 months with no history of treatment failure and no known resistance to its components	Yes *Notes:* Crushing not recommended in product information, however study data shows no significant effect on pharmacokinetics when crushed and administered with food or drip feed	EVG: CYP3A (major), UGT1A1/3 (minor)	1 tablet (EVG 150/COBI 150/FTC 200/TAF 10 mg) once daily PO with food	1	19 × 8.5	[28–31]
EVG 150 mg COBI 150 mg FTC 200 mg TDF 300 mg	Stribild^®^	Oral FDC tablet	HIV-1 Treatment: Adult and pediatric patients 12 years of age and older weighing > 35 kg with no ARV treatment history or to replace current ARV regimen in those with HIV-1 RNA < 50 copies/mL on a stable ARV regimen for at least 6 months with no history of treatment failure and no known resistance to its components	Yes *Notes:* Crushing not recommended in product information, however study data shows no significant effect on pharmacokinetics when crushed and administered with food or with drip feed		1 tablet (EVG 150/COBI 150/FTC 200/TDF 300 mg) once daily PO with food	1	20 × 10	[29–32]
RAL 400 mg	Isentress^®^	Oral tablet: 400 mg Chewable tablet: 25 mg 100 mg this is a third available dosage form for this product	HIV-1 Treatment: Adults and pediatric patients weighing > 2 kg in combination with other antiretroviral agents	Yes *Notes:* Crushing not recommended in product information, however study data shows acceptable plasma concentrations; important to note that alternative dosage forms available that may ease administration. Pediatric 25 mg chewable tablets may be crushed if swallowing is difficult	RAL: UGT1A1	Adults: 1 tablet (RAL 400 mg) twice daily PO with or without food Pediatric: Weight based dosing (refer to prescribing information)	2	16 × 8.8	[33]
RAL HD 600 mg	Isentress HD^®^	Oral tablet: 600 mg	HIV-1 Treatment: Adults and pediatric patients weighing > 40 kg in combination with other antiretroviral agents	No		2 tablets (RAL 600 mg × 2) once daily PO with or without food	2	19 × 9.7	[33]

*3TC*   lamivudine, *ABC*   abacavir, *BIC*  bictegravir, *CAB*  cabotegravir, *COBI*  cobicistat, *CYP3A* cytochrome P450 family 3 subfamily A, *DTG*  dolutegravir, *EVG*  elvitegravir, *FDC*  fixed dose combination, *FTC*  emtricitabine, *HD* high dose, *HIV-1*  human immunodeficiency virus type 1, *IM*  intramuscular, *INSTI*  integrase strand transfer inhibitor, *kg*  kilograms, *LA* long-acting, *mg*  milligrams, *mL*  milliliters, *N/A* not applicable, *PD* pediatric dose, *PO*  by mouth, *PrEP* pre-exposure prophylaxis, *RAL* raltegravir, *RPV* rilpivirine, *TAF*  tenofovir alafenamide, *TDF* tenofovir disoproxil fumarate, *UGT1A1* uridine disphosphate glucuronosyl transferase 1A1, *UGT1A9* uridine disphosphate glucuronosyl transferase 1A9

This article reviews current literature on the clinical pharmacology of INSTIs, discusses various topics relating to INSTI resistance, provides a comparative synopsis of INSTI characteristics, identification of clinically relevant DDIs associated with each INSTI, as well as summarizes major clinical trials evaluating efficacy and safety of INSTIs used in treatment-naive individuals living with HIV as well as HIV pre-exposure prophylaxis (PrEP). Finally, this review will provide important clinical considerations and guidance for individuals with HIV when using and choosing between various INSTIs. A discussion of the role of INSTIs in treatment-experienced individuals living with HIV is beyond the scope of this review and the reader is encouraged to refer to other published literature [34–40].

## Main text

### Clinical Pharmacology

#### Pharmacodynamics

All INSTIs have activity against HIV-1 isolated in human peripheral blood mononuclear cells, primary monocyte and macrophage cells, as well as CD4 + T-lymphocytes. Along with NRTIs and PIs, INSTIs also have activity against HIV-2. INSTIs act at the integration step of the retroviral replication cycle, this step being a hallmark of retroviruses and the point at which the proviral state is established, ensuring permanent infection. Integration is catalyzed by integrase, one of the three enzymes encoded in the HIV-1 genome [41]. Integrase appears to act within a large functional nucleoprotein complex that contains both viral DNA and many components derived from the virus and host cell. This complex is often referred to as the intasome. It is as part of the intasome that integrase catalyzes two reactions that are required to insert the reverse-transcribed HIV DNA into the DNA of the infected host cell.

The first reaction is referred to as the “3’-processing” step and the second a “strand transfer” reaction [41, 42]. During 3’-processing, integrase removes two or three nucleotides from each 3’ end of the viral DNA. This allows for the strand transfer reaction to occur, where after import of the viral DNA into the nucleus, integrase inserts the 3’ ends of the viral DNA into the host cell DNA. This integration process does not require any source of energy but requires divalent cations (such as manganese or magnesium) as cofactors for catalytic activity [43].

INSTIs can be generally characterized by a metal chelating scaffold positioned to bind two Mg^2+^ cofactors, a halogenated benzene side chain that interacts with viral DNA, and a flexible linker that connects the core scaffold to the halobenzyl side chain [44, 45]. INSTIs are understood to preferentially bind to the active site of integrase within the intasome rather than free integrase [41]. Interfering with the strand transfer reaction, the INSTI binds to the divalent cations and viral DNA, displacing the viral DNA from the active site and deactivating the intasome. Consequently, this blocking of the active site prevents integration of reverse-transcribed viral DNA into the host genome [42, 46].

Second generation INSTIs were developed with the intention of overcoming resistance observed in clinical practice with first generation INSTIs RAL and EVG, both of which have a high level of cross-resistance. Second generation INSTIs, DTG, BIC and CAB have less cross-resistance with first generation INSTIs, with DTG and BIC retaining the highest potency overall [44, 47]. It is from structural data of the INSTI-bound intasome that a greater understanding of the mode of INSTI binding has emerged, leading to chemical modifications to first-generation INSTIs and more potent compounds capable of overcoming known resistance patterns. Unlike first generation INSTIs, second generation compounds possess extended tricyclic scaffolds [48, 49]. For example, the core scaffold has been made larger in addition to attaching a third ring to increase the overall surface contact with the integrase active site, allowing for more binding stability. Additionally, the linker between the halobenzyl side chain and core scaffold was lengthened which allowed for a stronger interaction with the vDNA within the intasome [50].

An additional topic of interest lies within the differences in dissociation kinetics of various INSTIs at the molecular level. Studies investigating the second generation INSTIs DTG and BIC have shown considerably longer dissociation half-lives from the wildtype intasome complex as well as from mutant intasomes commonly observed during treatment with first generation INSTIs [51, 52]. The structural differences and prolonged binding of DTG and BIC to the intasome may contribute significantly to their durability against resistance mutations compared to first generation INSTIs [51–53].

#### Pharmacokinetics

Unlike the other INSTIs which are metabolized by both CYP3A4 and UGT1A1, RAL is primarily metabolized by hepatic glucuronidation mediated by UGT1A1 only. RAL is neither an inhibitor, inducer, nor substrate of CYP enzymes [9]. EVG is a major substrate of CYP3A and undergoes primarily oxidative metabolism by CYP3A and secondarily glucuronidation by UGT1A1/3 enzymes, and studies have shown that by coadministration with pharmacokinetic enhancers such as RTV or COBI, presystemic first-pass metabolism can be substantially reduced, allowing for maintenance of adequate trough concentrations and once daily dosing [11, 54]. DTG is primarily metabolized by UGT1A1 with minor contributions from CYP3A enzymes. It is a substrate of UGT1A1/3 and UGT1A9 enzymes including breast cancer resistance protein (BCRP) and P-glycoprotein (P-gp) drug transporters in vitro, and it inhibits organic cation transporter (OCT)2 and multidrug and toxin extrusion transporter (MATE)1 in vitro. Drugs that are inducers or inhibitors of these enzymes and transporters may affect drug concentrations of DTG [55, 56]. BIC is a substrate of CYP3A and UGT1A1 and similar to DTG, inhibits OCT2 and MATE1 in vitro, however is less potent in inhibiting OCT2 compared to DTG [57, 58]. CAB is primarily metabolized by UGT1A1 with minor contributions from UGT1A9. In vitro, it inhibits organic anion transporter (OAT)1 and OAT3 and may increase the area-under-the-curve (AUC) of medications that are substrates of OAT1/OAT3 by about 80% [59, 60]. A summary of pertinent drug-drug interactions between INSTIs and commonly prescribed comedications is included in Table 2. The reader is encouraged to refer to a recent comprehensive review for additional information related to the comparative clinical pharmacokinetics and pharmacodynamics of INSTIs [61].

**Table 2:** Summary of drug-drug interactions between INSTIs with selected coadministered drugs

Coadminstered Drug	INSTI	Effect on INSTI or Coadministered Drug Concentrations
Al, Mg, ± Ca-containing Antacids & Polyvalent Cation Supplements (Al, Ca, Fe, Mg, Zn, including multivitamins with minerals)	BIC	↓ BIC ⟷ BIC with food & Ca/Fe
	CAB	↓ CAB
	DTG	↓ DTG ⟷ DTG with food & Ca/Fe
	EVG/c	↓ EVG/c
	RAL	↓ RAL
**Alpha Adrenergic Inhibitors (for Benign Prostatic Hyperplasia)**		
Alfuzosin	EVG/c	↑ alfuzosin
Tamsulosin	EVG/c	↑ tamsulosin
Silodosin	EVG/c	↑ silodosin
**Anticoagulants & antiplatelets**		
Apixaban	EVG/c	↑ apixaban
Clopidogrel	EVG/c	↓ clopidogrel active metabolite, with impaired platelet inhibition expected
Dabigatran	EVG/c	↑ dabigatran
Edoxaban	EVG/c	↑ edoxaban
Prasugrel	EVG/c	↓ prasugrel active metabolite, with no impairement of platelet inhibition expected
Rivaroxaban	EVG/c	↑ rivaroxaban
Ticagrelor	EVG/c	↑ ticagrelor
Vorapaxar	EVG/c	↑ vorapaxar
Warfarin	EVG/c	↑ or ↓ warfarin
**Anticonvulsants**		
Carbamazepine	BIC	↓ BIC
	CAB	↓ CAB
	DTG	↓ DTG
	EVG/c	↑ carbamazepine, ↓ EVG & COBI
	RAL	↓ or ⟷ RAL
Eslicarbazepine	All INSTIs	↓ INSTI, ↓ COBI
Ethosuximide	EVG/c	↑ ethosuximide
Lamotrigine	EVG/c	No data
Oxcarbazepine	BIC, DTG	↓ BIC, DTG
	CAB	↓ CAB
	EVG/c, RAL	↓ EVG/c, RAL
Phenobarbital, phenytoin	BIC	↓ BIC
	CAB	↓ CAB
	DTG	↓ DTG
	EVG/c	↓ EVG/c
	RAL	↓ OR ⟷ RAL
Valproic Acid	All INSTIs	No data
**Antidepressants/ anxiolytics/ antipsychotics**		
Aripiprazole	EVG/c	↑ aripiprazole
Brexpiprazole	EVG/c	↑ brexpiprazole
Cariprazine	EVG/c	↑ cariprazine
Bupropion	EVG/c	↑ or ↓ bupropion
Buspirone	EVG/c	↑ buspirone
Fluvoxamine	EVG/c	↑ or ↓ EVG
Iloperidone	EVG/c	↑ iloperidone
Lumateperone	EVG/c	↑ lumateperone
Lurasidone	EVG/c	↑ lurasidone
Nefazodone	EVG/c	↑ nefazodone
Olanzapine	All INSTIs	⟷ olanzapine
Other antipsychotics (CYP3A4 and/or CYP2D6 substrates (e.g., perphenazine, risperidone, thioridazine)	EVG/c	↑ antipsychotic
Pimavanserin	EVG/c	↑ pimavanserin
Pimozide	EVG/c	↑ pimozide
Quetiapine	EVG/c	↑ quetiapine
SSRIs (citalopram, escitalopram, fluoxetine, fluvoxamine, paroxetine, sertraline)	BIC, CAB, DTG, RAL	⟷ SSRI
	EVG/c	⟷ sertraline, ↑ other SSRI
TCAs (amitriptyline, desipramine, doxepin, imipramine, nortriptyline)	EVG/c	↑ TCAs
Trazodone	EVG/c	↑ trazodone
Ziprasidone	EVG/c	↑ ziprasidone
**Antifungals**		
Isavuconazole	EVG/c	↑ isavuconazole ↑ or ↓ EVG & COBI
Itraconazole, Posaconazole, Voriconazole	EVG/c	↑ antifungal ↑ EVG & COBI
**Antihyperglycemic drugs**		
Metformin	BIC	↑ metformin
	DTG	↑ metformin
	CAB PO and IM, RAL	⟷ metformin
Saxagliptan	EVG/c	↑ saxagliptan
**Antibacterials**		
Azithromycin	All INSTIs	⟷ azithromycin
Clarithromycin	EVG/c	↑ clarithromycin, COBI
Erythromycin	EVG/c	↑ erythromycin, COBI
Rifabutin	BIC	↓ BIC
	CAB	↓ CAB IM
	DTG	⟷ DTG
	EVG/c	↑ rifabutin active metabolites, ↓ EVG
	RAL	↑ RAL
Rifampin	BIC	↓ BIC
	CAB	↓ CAB
	DTG	↓ DTG
	EVG/c	↓ EVG & COBI
	RAL	↓ RAL
Rifapentine	BIC	↓ BIC
	CAB	↓ CAB
	DTG	↓ DTG
	EVG/c	↓ EVG & COBI
	RAL	↑ or ↓ RAL
**Cardiac medications**		
Amiodarone	EVG/c	↑ amiodarone
Beta-blockers (i.e., metoprolol, timolol, etc.)	EVG/c	↑ beta-blockers
Calcium channel blockers (CCBs)	EVG/c	↑ CCB
Dofetilide	BIC, DTG	↑ dofetilide
	EVG/c	↑ dofetilide
Eplerenone	EVG/c	↑ eplerenone
**Herbal products**		
St. John’s Wort	BIC, CAB, DTG	↓ BIC & DTG
	EVG/c	↓ EVG & COBI
**Hormonal therapies**		
Contraceptives: non-oral	BIC, CAB, DTG, RAL	Etonogestrel (subdermal implant) ↑ with DTG ⟷ BIC, CAB, RAL
Contraceptives: oral	BIC, CAB, DTG, RAL	⟷ ethinyl estradiol, norgestimate, ⟷ levonorgestrel with PO CAB
	EVG/c	↑ norgestimate, drospirenone ↓ ethinyl estradiol
Gender-Affiriming Therapy	EVG/c	↑ estradiol, cyproterone, dutasteride, & finasteride ↑ testosterone
Menopausal Hormone Replacement Therapy	BIC, CAB, DTG, RAL	↓ estrogen from conjugated estrogen (equine or synthetic) or estradiol ⟷ drospirenone, medroxyprogesterone, or micronized progesterone
	BIC, CAB, EVG/c	↓ or ↑ estrogen, ↑ drospirenone, PO medroxyprogesterone, PO micronized progesterone
**Immunosuppressive drugs**		
Cyclosporine, everolimus, sirolimus, tacrolimus	EVG/c	↑ immunosuppressive therapy
**Lipid-modifying agents**		
Atorvastatin	EVG/c	↑ atorvastatin
Lomitapide	EVG/c	↑ lomitapide
Lovastatin	EVG/c	↑ lovastatin
Pitavastatin, pravastatin	EVG/c	No data
Rosuvastatin	EVG/c	↑ rosuvastatin
Simvastatin	EVG/c	↑ simvastatin
**Narcotics/ opioid dependence treatment**		
Buprenorphine (Sublingual, buccal, or implant)	EVG/c	↑ buprenorphine, norbuprenorphine
	RAL	⟷ sublingual, implant
Fentanyl	EVG/c	↑ fentanyl
Lofexidine	EVG/c	↑ lofexidine
Methadone	BIC, CAB, DTG, EVG/c, RAL	No significant effect
Tramadol	EVG/c	↑ tramadol, ↓ M1 (active metabolite)
**PDE-5 inhibitors**		
Avanafil	EVG/c	No data
Sildenafil	EVG/c	↑ sildenafil
Tadalafil	EVG/c	↑ tadalafil
Vardenafil	EVG/c	↑ vardenafil
**Sedatives/hypnotics**		
Alprazolam, clonazepam, clorazepate, diazepam, estazolam, flurazepam	EVG/c	↑ benzodiazepines
Midazolam, triazolam	DTG	⟷ midazolam
	EVG/c	↑ midazolam, triazolam
Suvorexant	EVG/c	↑ suvorexant
Zolpidem	EVG/c	↑ zolpidem

*BIC* bictegravir, *CAB* cabotegravir, *COBI* cobicistat, *DTG* dolutegravir, *EVG/c* elvitegravir/cobicistat, *IM* intramuscular, *INSTI* integrase strand transfer inhibitor, *PO* by mouth, *RAL* raltegravir [2, 10–12, 119, 120]

#### Resistance

##### Resistance within integrase region

Historically, resistance mutation development to INSTIs has focused on the involvement of well-identified mutations in the target gene, integrase. The basic mechanism of resistance within integrase is well described and generally begins with an initial mutation reducing the binding affinity of the drug, but also often reduces the fitness of the virus. Continued selective drug pressure can lead to development of secondary resistance substitutions that ultimately may improve viral fitness, resulting in an increased level of INSTI resistance [62].

For first generation INSTIs, amino acid substitutions at either Tyr143 (Y143H/R/C) or Gln148 (Q148/H/R/K) or N155H plus one or more additional substitutions (i.e., L74M, E92Q, Q95K/R, T97A, E138A/K, G140A/S, V151I, G163R, H183P, Y226C/D/F/H, S230R, and D232N) leads to RAL resistance [63, 64]. Primary integrase substitutions T66A/I, E92G/Q, S147G, and Q148R cause reduced susceptibility to EVG [65–69]. E92Q is the most common initial mutation that is seen with failure on EVG-based regimens, followed by N155H and Q148H/R/K [70]. In the presence of E92Q, high-level resistance is conferred to EVG and intermediate-level resistance is conferred to RAL [71]. For individuals with EVG or RAL mutations, some of these INSTI mutations can be overcome by using twice daily dosing of DTG rather than its standard once daily dosing in combination with an optimized background regimen [40].

For second generation INSTIs, amino acid substitutions E92Q, G118R, S153F/Y, G193E, R263K decrease DTG susceptibility two- to four-fold [72]. M50I tends to be selected in vitro by DTG and BIC in combination with R263K, contributing to reduced DTG susceptibility [72]. The R263K, E92Q, Y143R, N155H, and Q148R substitutions confer 1.8-, 1.2-, 1.1-, 1.0- and 0.7-fold reduced susceptibility to BIC in vitro, respectively [73].

In clinical trials and in clinical practice, INSTI resistance is uncommon. One study evaluated integrase genotyping results of over 3,000 individuals between 2009 and 2012 and found that 15.6% of individuals had viruses with one or more integrase major mutations, most mutations being either N155H or Q148H/K/R [70]. Resources such as the HIV Stanford Database should be referred to frequently as interpretation of susceptibilities of these INSTIs to various polymorphisms may change as newer data become available [71, 74]. Refer to Table 3 for a list of major INSTI resistance associated mutations (RAMs) and how these affect the susceptibility of each INSTI. This table was created based on the Drug Resistance Mutation (DRM) penalty scoring system utilized by the HIV Stanford Database. The reader is encouraged to refer to the HIVdb program within the HIV Stanford Database for additional details regarding DRM penalty scoring [71].

**Table 3:** Relative Resistance of INSTI Resistance Associated Mutations (RAMs)

RAMs	INSTI				
	RAL	EVG	CAB	DTG	BIC
Y143R/C/H/S	+ + + +	+	+	−	−
S147G	+	+ + + +	+	+	+
E138K/A/T	+ +	+ +	+	+	+
G140S/A/C	+ + +	+ + +	+	+	+
E92G/Q	+ + +	+ + + +	+ +	+	+
N155H	+ + + +	+ + + +	+ +	+	+
F121Y	+ + + +	+ + + +	+ +	+	+
T66A/I/K	+ + + +	+ + + +	+ +	+ +	+ +
Q148H/R/K	+ + + +	+ + + +	+ + +	+ + +	+ + +
R263K	+ +	+ + +	+ + +	+ + +	+ + +
G118R	+ + + +	+ + + +	+ + + +	+ + +	+ + +

− susceptible^a^ |+ potential low-level resistance^b^ |+ + low-level resistance^c^ |+ +  + intermediate resistance^d^ |+ +  +  + high-level resistance^e^

^a^Susceptible: no evidence of reduced ARV susceptibility compared with wild-type virus

^b^Potential low-level resistance: sequence may contain mutations indicating previous ARV exposure or many contain mutations that are associated with drug resistance only when they occur with additional mutations

^c^Low-level resistance: Virus encoded by specific sequence may have reduced in vitro ARV susceptibility, or patients harboring viruses with the submitted mutations may have a suboptimal virological response to treatment

^d^Intermediate resistance: High likelihood that drug’s activity will be reduced, but the drug will likely retain significant antiviral activity

^e^High-level resistance: predicted level of resistance is like those observed in viruses with the highest levels of in vitro drug resistance, or clinical data exist demonstrating patients typically have little or no virologic response to treatment with the ARV [71]

Even with the structural improvements to second generation INSTIs, it is evident by their resistance profiles described above that these compounds are still susceptible to viral resistance. Recent studies have investigated utilization of red-capped mangabey simian immunodeficiency (SIV) integrases to better visualize the mode of INSTI binding within the intasome and thus better understand generation of INSTI resistance at this important site. Findings from such studies suggest that amino acid substitutions resulting in INSTI resistance are driven by extremely sensitive magnesium ion binding geometry within the active site, a phenomenon that the virus can manipulate for its own survival benefit [48, 75]. In this regard, the dependence of INSTIs on metal ion coordination is both their strength and weakness. Studies examining interactions between INSTIs and the HIV/SIV intasome propose that further extending the INSTI scaffolds into “free spaces” toward the integrase backbone in a manner that creates more contacts within the active site is a concept that should be explored to stabilize binding geometry and thus improve the ability of new INSTIs to overcome potential resistance [44, 48, 75]. A structural modification that has shown promise in retaining potency against known mutant variant combinations is the development of compounds containing various modifications of a naphthyridine core scaffold. In general, these compounds tend to bind closer to integrase in the active site, supporting their ability to avoid development of viral RAMs [45].

##### Resistance outside of integrase region

Virologic failure to second generation INSTIs has occurred in patients who had no selected mutations in the integrase gene [76–78]. Such findings have suggested that HIV-1 can use alternative mechanisms to develop resistance to INSTIs outside of the integrase gene. One of the two more studied methods outside of integrase by which HIV-1 has developed resistance to INSTIs is by changes to the 3-prime polypurine tract (3’-PPT). The 3’-PPT is a primer required during the process of reverse transcription from HIV-1 RNA into double-stranded DNA. Malet et al. reported that following in vitro selection with high plasma concentrations of DTG, an HIV-1 strain showed no mutations in integrase, but multiple mutations in the 3’-PPT [79]. Wijting et al. reported similar findings in vivo with a patient failing dolutegravir maintenance monotherapy who developed mutations in the 3’-PPT and not in the integrase gene [80]. However, other studies suggest that mutations in the 3’-PPT in individuals failing INSTI treatment may not necessarily contribute to INSTI resistance [81]. Recent findings suggest there may be an explanation for resistance to INSTIs in this setting; through further *in-vitro* investigation of the mutations in 3’-PPT noted by Malet et al., DTG did not impair the replication of these mutants and no integrated viral DNA could be detected. Instead, accumulation of unintegrated viral DNA was observed [82]. Previous studies have shown that unintegrated viral DNA is involved in the expression of some early viral proteins and under certain conditions, unintegrated viral DNA has led to low-level viral replication [83, 84]. These findings highlight the important role of unintegrated viral DNA in overall viral replication in patients treated with INSTIs and may help explain why some patients experience INSTI treatment failure while failing to develop integrase RAMs. Additional studies are warranted to better understand the mechanism by which mutations in the 3’-PPT may play a role in causing resistance to second generation INSTIs.

The second means by which INSTI resistance has been observed outside of the integrase gene is via mutations in the HIV-1 envelope glycoprotein complex (*env*). To describe this method of resistance acquisition, it is useful to first understand the role of *env* in productive viral transmission. Infectious particles that can lead to spread of infection are generated from the expression of *env* on membranes of both free virions and infected cells [85]. Two glycoprotein (gp) subunits of *env* are gp120 and gp41. Once gp120 binds to CD4 on the target cell surface, a conformational change is triggered in *env*, exposing the chemokine coreceptor (CCR5 or CXCR4)-binding site which allows for gp41-mediated fusion of the viral and host cell membranes [86]. Viral transmission from infected to uninfected cells can occur via two methods: cell-free infection or cell–cell transmission at points of cell–cell contact known as virological synapses. The virological synapses are formed by the interaction of *env* of the infected cell with the CD4 receptor on the target cell [86, 87]. Transmission occurring via the cell–cell route is significantly more effective than cell-free infection, leading to a higher multiplicity of infection (MOI) and thus a greater likelihood of overcoming ARV activity, among other barriers to infection [88].

In recent studies, investigation into treatment failure with INSTIs without emergence of INSTI RAMs has led to discoveries of mutations to the *env*-coding region [85, 89]. These *env* mutations appear to allow for an even greater efficiency of transmission between cells and suggest that it is possible for HIV-1 to avoid inhibitory activity of all ARVs via mutations in *env*. Interestingly, Van Duyne and colleagues observed that the calculated fold-change for certain *env* mutations is comparable to multiple integrase mutants in patients experiencing virologic failure with DTG [74, 85]. Like other mechanisms outside of integrase that confer resistance to INSTIs, further research is needed to elucidate the role of this mechanism of drug resistance and the impact of *env* mutations on the effectiveness of ARVs.

### Clinical studies involving efficacy and safety and tolerability of INSTIs in treatment-naïve individuals living with HIV

A summary of the clinical efficacy and safety of first generation INSTIs RAL and EVG at week 48 can be found in Tables 4 and 5, respectively, for convenience of viewing and comparing to second generation INSTIs. For the purposes of this review, second generation INSTIs will be discussed in more detail below.

**Table 4:** First Generation INSTIs: Virologic Outcomes at Week 48 (FDA Snapshot)

	STARTMRK		ONCEMRK		GS-US-236–0102		GS-US-236–0103		GS-US-292–0104/0111	
	RAL + FTC/TDF n = 281	EFV + TDF/ FTC n = 282	RAL 1200 QD + TDF/ FTC n = 531	RAL 400 BID + TDF/ FTC n = 266	EVG/ COBI/ FTC/ TDF n = 348	EFV/FTC/TDF n = 352	EVG/ COBI/ FTC/ TDF n = 353	ATV/RTV + FTC/ TDF n = 355	EVG/ COBI/ FTC/ TAF n = 866	EVG/ COBI/ FTC/ TDF n = 867
HIV- 1 RNA < 50 copies/mL	241 (86.1%)	230 (81.9%)	472 (89%)^a^	235 (88%)^a^	305 (87.6%)	296 (84.1%)	316 (89.5%)	308 (86.8%)	800 (92%)	784 (90%)
Difference, % (95% CI)	4.2 (−1.9, 10.3)		−0.5 (−4.2, 5.2)		3.6 (−1.6, 8.8)		3.0 (−1.9, 7.8)		2.0 (−0.7, 4.7)	
HIV- 1 RNA ≥ 50 copies/mL	27 (9.6%)	39 (13.8%)	29 (5%)	16 (6%)	25 (7.2%)	25 (7.1%)	19 (5.4%)	19 (5.4%)	–	–
No virologic data	–	–	30 (6%)	15 (6%)	18 (5.2%)	31 (8.8%)	18 (5.1%)	28 (7.9%)	34 (4%)	52 (6%)
INSTI resistance mutations detected	E92Q + L74L/M + T97T/A + Y143Y/H; G140S + Q148H/R; Y143R	None	E92Q + L74M; N155H; N155H + V151I; N155H + I203M	None	E92Q; N155H; Q148R; T66I	None	E92Q + N155H + T66I; N155H; Q148R	None	E92Q N155H Q148R + T66I/A T66A	E92Q E92Q + Q148R Q148R
References	[90]		[63]		[91]		[11]		[65]	

Dash (-) indicates that information was not reported in the study results

*3TC* lamivudine, *ABC* abacavir, *ATV* atazanavir, *BIC* bictegravir, *CI* confidence interval, *COBI* cobicistat, *DTG* dolutegravir, *EFV* efavirenz, *EVG* elvitegravir, *FDA* food and drug administration, *FTC* emtricitabine, *INSTI* integrase strand transfer inhibitor, *TAF* tenofovir alafenamide, *TDF* tenofovir disoproxil fumarate, *NRTI* nucleoside reverse transcriptase inhibitor, *RAL* raltegravir, *RTV* ritonavir

^a^HIV-1 RNA reported as HIV-1 RNA < 40 copies/mL

**Table 5:** First Generation INSTIs: Selected drug-related adverse events after week 48 recorded in 2% or more patients in either treatment group

	Raltegravir(RAL + FTC/TDF)				Elvitegravir(EVG/COBI/FTC/TDF)					
	STARTMRK^a^		ONCEMRK^a^		GS-236-0102^b^		GS-236-0103^b^		GS-292–0104/0111^d^	
Adverse Reaction	RAL n = 281	EFV n = 282	RAL 1200 mg QD n = 531	RAL 400 mg BID n = 266	EVG/ COBI FTC/ TDF n = 348	EFV/ FTC/ TDF n = 352	EVG/ COBI/ FTC/ TDF n = 353	ATV/ RTV + FTC/ TDF n = 355	EVG/ COBI/ FTC/ TAF N = 866	EVG/ COBI/ FTC/ TDF N = 867
Diarrhea	1%	3%	2%	3%	23%	19%	22%	27%	17%	19%
Nausea	3%	4%	7%	7%	21%	14%	20%	19%	15%	17%
Vomiting	-	-	2%	1%	-	-	-	-	7%	6%
Fatigue	1%	3%	-	-	11%	13%	14%	13%	8%	8%
Upper respiratory infection	-	-	-	-	14%	11%	15%	16%	11%	13%
Dizziness	1%	6%	2%	3%	7%	24%	-	-	5%	4%
Headache	4%	5%	3%	5%	14%	10%	15%	12%	14%	13%
Insomnia	4%	3%	-	-	9%	14%	-	-	7%	6%
Depression	-	-	-	-	9%	11%	-	-	-	-
Rash	-	-	-	-	6%	12%	-	-	6%	5%
Discontinuations due to drug-related clinical adverse events	1%	4%	0%	1%	4%^c^	5%^c^	4%^c^	5%^c^	1%	1%
References	[90]		[63]		[91]		[11]		[65]	

Dash (-) indicates that information regarding this adverse effect was not reported in the study results

*ATV* atazanavir, *COBI* cobicistat, *EFV* efavirenz, *EVG* elvitegravir, *FTC* emtricitabine, *RAL* raltegravir, *RTV* ritonavir, *TDF* tenofovir disoproxil fumarate

^a^Reported as drug-related clinical adverse events of moderate-severe intensity

^b^Reported as adverse events in ≥ 10% of patients in either group

^c^Discontinuations reported as patients with any treatment-emergent adverse event leading to premature discontinuation of study drug; whether they were deemed to be drug-related was not distinguished

^d^Reported as adverse events in ≥ 5% of patients in either group

#### Dolutegravir clinical efficacy

The clinical efficacy and safety of DTG were evaluated in three landmark phase 3 randomized, double-blind, active-controlled trials [6, 92, 93]. Refer to Table 6 for a summary of virologic efficacy data of all three trials at week 48. The first trial, SPRING-2, was the first head-to-head comparison of two INSTI-based regimens for first-line ART, comparing once daily DTG versus twice daily RAL in treatment-naïve individuals [92]. By week 48 of the 20 individuals (5%) in the DTG group and 28 (7%) in the RAL group that had protocol-defined virologic failure (PDVF), no individuals in the DTG group and one in the RAL group developed INSTI resistance. By week 96, 81% of individuals in the DTG treatment group and 76% in the RAL treatment group had HIV-1 RNA < 50 copies/mL [6]. Through follow-up at week 96, two additional individuals in the DTG group and one in the RAL group developed PDVF; however, no additional INSTI resistance was detected in either treatment group [6, 92].

**Table 6:** Second Generation INSTIs: Virologic Outcomes at Week 48 (FDA Snapshot)

	SPRING-2		SINGLE		GEMINI-1 and -2		GS-US-380–1489		GS-US-380–1490		FLAIR		ATLAS	
	DTG + 2 NRTIs n = 411	RAL + 2 NRTIs n = 411	DTG + ABC/ 3TC n = 414	EFV/ FTC/ TDF n = 419	DTG/ 3TC n = 716	DTG + FTC/ TDF n = 717	DTG/ABC/3TC n = 315	BIC/ FTC/ TAF n = 314	DTG + FTC/ TAF n = 325	BIC/ FTC/ TAF n = 320	CAB n = 283	CAR n = 283	CAB n = 308	CAR n = 308
HIV-1 RNA < 50 copies/mL	361 (88%)	351 (85%)	364 (88%)	338 (81%)	655 (91%)	669 (93%)	293 (93%)	290 (92%)	302 (93%)	286 (89%)	265 (94%)	264 (93%)	285 (93%)	294 (95%)
Difference, % (95% CI)	2.5 (−2.2, 7.1)		7.4 (2.5, 12.3; *P* = 0.003)		−1.7 (−4.4, 1.1)		−0.6 (−4.8, 3.6; *P* = 0.78)		−3.5 (−7.9, 1; *P* = 0.12)		−0.4 (−2.8, 2.1)		0.7 (−1.2, 2.5)	
HIV-1 RNA ≥ 50 copies/mL	8 (2%)	5 (1%)	6 (1%)	5 (1%)	8 (1%)	5 (1%)	8 (2.5%)	3 (1%)	4 (1%)	14 (4%)	6 (2%)	7 (2%)	5 (2%)	3 (1%)
No virologic data	30 (7%)	29 (7%)	29 (7%)	55 (13%)	41 (6%)	35 (5%)	14 (4%)	21 (7%)	19 (6%)	20 (6%)	12 (4%)	12 (4%)	18 (6%)	11 (4%)
INSTI Resistance Mutations Detected	None	T97T/A, E138E/D, V151V/I, N155H	None	N/A	None	None	None	None	None	None	Q148R; G140R	None	N155H; N155H + R263K	None
Reference	[92]		[93]		[96]		[97]		[98]		[99]		[100]	

Dash (-) indicates that information was not reported in the study results

*3TC* lamivudine, *ABC* abacavir, *ATV* atazanavir, *BIC* bictegravir, *CAR* current antiretroviral regimen, *CI* confidence interval, *COBI* cobicistat; *DTG* dolutegravir, *EFV* efavirenz, *EVG* elvitegravir, *FTC* emtricitabine, *NRTI* nucleoside reverse transcriptase inhibitor, *RTV* ritonavir, *RAL* raltegravir, *TAF* tenofovir alafenamide, *TDF* tenofovir disoproxil fumarate

The second trial, SINGLE, compared the efficacy of DTG with either ABC/3TC or FTC/TDF to efavirenz (EFV)/FTC/TDF in treatment-naïve individuals [93]. By week 48, of the 18 (4%) individuals in the DTG group that met criteria for PDVF (defined as two consecutive HIV-1 RNA values of > 50 copies/mL on or after week 24) and had resistance testing performed, only two had more than low-level viremia (HIV-1 RNA of > 200 copies/mL), with no integrase RAMs detected [93]. Week 96 results showed 80% of individuals in the DTG group and 72% in the EFV group had HIV-1 RNA < 50 copies/mL [8]. Through week 144, 71% of individuals in the DTG group and 63% in the EFV group achieved virologic suppression of HIV-1 RNA < 50 copies/mL [8]. Through week 144, among 39 (9%) of individuals who met criteria for PVDF, 29 of these had low-level viremia with no integrase RAMs detected in the DTG group [94].

The third trial, GEMINI−1 and GEMINI−2 were duplicate non–inferiority studies in treatment−naïve adult individuals infected with HIV−1 comparing the two−drug (2DR) regimen DTG plus 3TC to the three−drug regimen (3DR) of DTG plus FTC/TDF. By week 48, 10 (< 1%) individuals met criteria for confirmed virologic failure (six in the 2DR group and four in the 3DR group) with all these individuals discontinuing the study. No treatment-emergent resistance to any component was reported in either treatment group in both clinical trials. At week 96, 86% and 90% of individuals maintained virologic suppression in the treatment groups, respectively [95]. Furthermore, through week 144, 82% and 84% of individuals maintained virologic suppression, respectively [96]. Through week 144, a total of 12 individuals (1.7%) in the 2DR group and 9 (1.3%) in the 3DR group met criteria for confirmed virologic withdrawal. Of these, none had treatment emergent INSTI RAMs. One patient in the 2DR group did not meet confirmed virologic withdrawal study criteria but developed R263R/K integrase mutation and had an HIV-1 RNA of 135 copies/mL at week 144. This patient was later resuppressed with a protease-inhibitor and INSTI-based regimen [96].

#### Dolutegravir safety and tolerability

Refer to Table 7 for a summary of virologic safety data of all three trials at week 48. In SPRING-2 study, rates of adverse events leading to discontinuation were low. Increases in serum creatinine were observed in both treatment groups by week two and stabilized thereafter, and none of these cases resulted in treatment discontinuation. Rates of adverse events continued to be similar through week 96 with no additional subjects discontinuing treatment with DTG due to adverse events [76].

**Table 7:** Second Generation INSTIs: Selected drug-related adverse events after week 48 recorded in 2% or more patients in either treatment group

	Dolutegravir						Bictegravir					Cabotegravir	
	SPRING-2^a^		SINGLE		GEMINI-1 and -2^b^ – Pooled Analysis		GS-US-380–1489^a^		GS-US-380–1490^a^			FLAIR and ATLAS – Pooled Analysis	
Adverse reaction	DTG n = 411	RAL n = 411	DTG n = 414	EFV n = 419	DTG/3TC n = 716	DTG and FTC/ TDF n = 717	DTG n = 315	BIC n = 314		DTG n = 325	BIC n = 320	CAB n = 591	CAR n = 591
Diarrhea	11%	11%	17%	18%	9%	11%	13%	13%		12%	12%	–	–
Nausea	14%	13%	14%	14%	4%	7%	23%	10%		9%	8%	3%	1%
Vomiting	–	–	5%	5%	–	–	5%	4%		–	–	–	–
Fatigue	5%	4%	13%	12%	-	-	9%	6%		8%	6%	5%	< 1%
Upper respiratory infection	6%	6%	9%	10%	8%	6%	11%	6%		7%	5%	–	–
Dizziness	6%	6%	9%	35%	–	–	–	–		1%	2%	2%	< 1%
Headache	12%	12%	13%	13%	10%	10%	14%	11%		12%	13%	4%	< 1%
Insomnia	5%	4%	15%	10%	4%	6%	6%	4%		4%	5%	–	–
Depression	5%	3%	6%	6%	–	–	–	–		–	–	-	–
Rash	–	–	3%	14%	–	–	–	–		–	–	2%	0%
Pyrexia	–	-	-	-	-	-	–	–		–	–	8%	0%
Injection site reactions	–	–	–	–	–	-	–	–		-	–	83%	0%
Discontinuations due to drug-related clinical adverse events	2%	2%	2%	10%	1%	1%	1%	0%		< 1%	2%	80%	–
References	[92]		[93]		[96]		[97]			[98]		[99, 100]	

Dash (-) indicates that information regarding this adverse effect was not reported in the study results or was reported as < 2% for both groups

*3TC* lamivudine, *BIC* bictegravir, *CAB* cabotegravir, *CAR* current antiretroviral regimen, *DTG* dolutegravir, *EFV* efavirenz, *FTC* emtricitabine, *RAL* raltegravir, *RPV* rilpivirine, *TDF* tenofovir disoproxil fumarate

^a^Adverse events reported as adverse events with ≥ 5% incidence in either group

^b^Adverse events reported as adverse events with ≥ 4% incidence in either group

In the SINGLE trial, primary reasons for discontinuation of DTG were psychiatric disorder (n = 2) and skin and subcutaneous-tissue disorder (n = 2). Similar to the SPRING-2 trial, small increases in serum creatinine of 0.12 to 0.15 mg/dL were observed in DTG group by week two, stabilizing through week 48. [92, 93]. Tolerability and safety of both treatment groups continued to be similar through week 144. No clinically significant differences in abnormal laboratory parameters were observed between weeks 48 and 144 [94].

In the GEMINI-1 and -2 trials, reasons for discontinuation were not described. Changes in renal biomarkers and most of the bone turnover biomarkers significantly favored the 2DR, with mean change in these biomarkers from baseline significantly greater in the 3DR. At week 48, HDL increased significantly in the 2DR compared to the 3DR. These changes in lipid parameters are considered consistent with the anticipated effect of TDF on lipids [101]. Through week 144, the main reasons for discontinuation were psychiatric disorders (n = 11 [2DR], n = 8 [3DR]), renal related (n = 2 [2DR], n = 12 [3DR])), and osteoporosis (none in 2DR and two in 3DR). Tolerability and safety observed in both treatment groups continued similarly through week 144, with comparable rates of adverse events of all grades across both treatment groups [96]. Of note, weight gain was noted in the study results of this trial and was observed in 2% of individuals per group; this led to discontinuation in one patient in the 2DR. The mean change in weight from baseline to week 144 was 3.7 kg in the 2DR and 2.4 kg in the 3DR. Through week 144, changes in renal biomarkers continued to favor the 2DR group, increases in bone turnover from baseline continued to be lower for the 2DR group, and lipid parameters generally favored the 3DR, which was consistent with the 48 week findings.

#### Bictegravir clinical efficacy

The clinical efficacy and safety of a FDC of BIC/FTC/TAF versus a DTG-based, 3-drug regimen in treatment-naïve individuals were assessed in two landmark phase 3 randomized, double-blind, active-controlled trials [97, 98]. Refer to Table 6 for a summary of virologic efficacy data of both trials at week 48. In the GS-1489 clinical trial, the efficacy of the FDC of BIC/FTC/TAF compared to the FDC of DTG/ABC/3TC as initial treatment of HIV-1 was evaluated [97]. The durability of the 48-week results was observed through week 96 (88% versus 90%) and week 144 (82% versus 84%) in both treatment groups, respectively [97, 102]. By week 48, one (< 1%) patient in the BIC group and four (1.3%) in the DTG group met protocol-defined criteria for resistance testing. Of these, none had treatment emergent resistance.

The second trial, GS-1490, evaluated the efficacy of BIC/FTC/TAF compared to DTG plus FTC/TAF as initial treatment of HIV-1 infection [98]. By week 48, seven (2.1%) individuals in the BIC group and five (1.5%) in the DTG group met criteria for resistance testing, however no treatment resistance to any component of either regimen was observed [98]. Through week 144 as a composite analysis of both studies 1489 and 1490, a total of six (2%) individuals in the DTG group and none in the BIC group met criteria for resistance testing; no resistance was seen to any component of either treatment group [102].

#### Bictegravir safety and tolerability

Refer to Table 7 for a summary of virologic safety data of both trials at week 48. In the GS-1489 study, reasons for discontinuation of DTG/ABC/3TC were nausea and generalized rash (n = 1), thrombocytopenia (n = 1), chronic pancreatitis and steatorrhea (n = 1), and depression (n = 1). Grade 3 or 4 laboratory abnormalities were reported in 15% of individuals in both arms and were similar across each group. At week 144, adverse events and laboratory abnormalities were similar between both treatment groups [102].

In the GS-1490 study, the three discontinuations in the BIC group deemed related to the study drug at week 48 were due to chest pain (n = 1), abdominal distension (n = 1), sleep disorder, dyspepsia, tension headache, depressed mood, and insomnia (n = 1). The discontinuation in the DTG group was considered unrelated to the study drug [98]. Grade 3 or 4 laboratory abnormalities were reported in 17% of individuals in the BIC group and 13% of individuals in the DTG group; trends in these abnormalities were not observed across either group. At week 144, adverse events leading to discontinuation were reported similarly in both groups with six individuals (2%) in each [102].

#### Cabotegravir clinical efficacy

The clinical efficacy and safety of CAB were evaluated in two landmark phase 3 randomized, double-blind, active-controlled trials [99, 100]. Refer to Table 6 for a summary of virologic efficacy data of both trials at week 48. Because of CAB’s unique long-acting (LA) formulation, the study design for these comparison trials is different from its counterparts in the INSTI class; therefore, additional details have been included below.

The first trial, FLAIR, was a phase 3 randomized, open-label, non-inferiority trial designed for individuals naïve to HIV treatment. The trial included three main phases: induction, maintenance, and extension. The induction phase consisted of daily oral DTG/ABC/3TC for 20 weeks to reach virologic suppression, at which point individuals were randomized 1:1 to start maintenance phases of either oral lead-in therapy of the study drugs or continue oral therapy of DTG/ABC/3TC. Oral lead-in therapy consisted of CAB 30 mg and RPV 25 mg once daily for approximately four weeks before initiating a loading dose of LA CAB 600 mg (3 mL) and RPV 900 mg (3 mL) IM, followed by monthly IM injections of CAB 400 mg (2 mL) and RPV 600 mg (2 mL), with the maintenance phase designed to last for 96 weeks. The extension phase was offered to participants in the oral-therapy group who maintained viral suppression through week 96, which meant they could switch to long-acting therapy at this time [99].

Results from the study notably showed that at week 48, 91% of study participants preferred LA therapy [99]. By week 48, four participants in the long-acting therapy group had confirmed virologic failure with three cases developing NNRTI and INSTI RAMs during treatment. These participants all had L74I integrase mutation at baseline; however, 51 of the 54 individuals in the long-acting group who had this mutation at baseline did not have subsequent virologic failure. Through week 96, results remained consistent with no individuals developing virologic failure between week 48 and 96 [103].

The second trial, ATLAS, was a phase 3, open-label, multicenter noninferiority trial in which individuals who had plasma HIV-1 RNA levels of < 50 copies/mL for at least six months while taking a standard oral ARV regimen were either continued on their current regimen or switched to monthly IM injections of LA CAB and RPV [100]. After week 48, eligible participants could transition to the ATLAS-2 M study (investigating the same LA therapy but dosed every 8 weeks versus LA therapy dosed every 4 weeks) or enter the extension phase of ATLAS at week 52. In the extension phase, individuals either continued their LA every 4 weeks therapy or those who were originally randomized to their current ARV regimen were switched from that regimen to LA therapy every four weeks, this group being designated as the switch population [104].

Results from ATLAS at week 48 resulted in noninferiority criteria of the LA injectable being met. Of the individuals in the LA therapy group, three had confirmed virologic failure with no INSTI RAMs detected. Of note, there were no missed injections or late-administered injections for any patient experiencing virologic failure [100]. Of the participants who remained in the trial via the extension phase (through week 96), 100% (n = 23) of individuals originally in the LA group and 97% (n = 28/29) of individuals in the switch population maintained virologic suppression. Although no participant in either group met confirmed virologic failure criteria, one patient with HIV-1 RNA > 50 copies/mL at week 96 was placed on a protease-inhibitor based regimen for continued follow-up [104].

#### Cabotegravir safety and tolerability

Refer to Table 7 for a summary of virologic safety data of both trials at week 48. In the combined FLAIR and ATLAS pooled analyses, the primary reason for withdrawal, although minimal, was injection-site reactions (1%). Other reasons for withdrawal from treatment (incidence < 1%) were viral hepatitis, diarrhea, and headache. The median weight gain from baseline was 1.3 kg by week 48 in the long-acting therapy group compared to 1.0 kg in the comparator group. Grade 3 or 4 laboratory abnormalities observed most in the long-acting group were elevations in creatine kinase (9%), direct bilirubin (5%), and lipase (6%).

Through week 96 in the FLAIR study, discontinuations due to adverse events increased to 14 individuals (5%) in the long-acting group, with five of these considered drug-related. Reasons for withdrawal from treatment for these individuals were similar to the 48-week findings. Five additional individuals in the long-acting group discontinued treatment between 48 and week 96 for reasons such as depression, viral hepatitis, and injection site pain. By week 96, the median weight gain was 2.0 kg in both the long-acting group and similarly in the standard care group. The most commonly observed laboratory abnormalities in the long-acting group did not differ from week 48 results [103].

### Clinical studies involving efficacy and safety and tolerability of cabotegravir for pre-exposure prophylaxis of HIV

#### Clinical efficacy

The clinical efficacy and safety of LA CAB as PrEP of HIV-infection has been evaluated in two phase 3 randomized, double-blind, multicenter international studies, HPTN-083 and HPTN-084, and the nature of these studies are described below [105, 106].

Both HPTN-083 and HPTN-084 trials included the same three phases: oral-tablet lead-in phase, an injection phase, and a “tail phase.” In both treatment groups, all participants were given an oral daily active study drug, either of CAB or FTC/TDF as a fixed dose, for up to five weeks to demonstrate patient tolerability**.** To initiate the injection phase, all participants were given 3-ml IM injections at months 1 and 2, then every eight weeks thereafter for a period of 153 weeks along with a placebo (either injection or oral tablet) of the alternative treatment. The tail phase included monitoring beginning eight weeks after the final injection and continuing for approximately 48 weeks [105, 106].

The first trial, HPTN-083, was designed for cisgender men and transgender women (TGW) who have sex with men. Results from the study showed that at week 153, 12 (0.37%) participants in the CAB group versus 39 (1.22%) participants in the FTC/TDF group had acquired incident HIV-1 infection (hazard ratio 0.31 [95% CI 0.16–0.58])**.** The low incidence rate in both arms demonstrated that both study drug agents were effective in preventing HIV. Of the 12 individuals that developed incident infection, INSTI RAMs were detected in 4 participants and included the following: E138A, E138E/K, E157Q, G140A, G140G/S, L74I, Q148R, and R263K**.** Results from the study suggest that CAB LA yielded a 69% lower risk in acquiring HIV infection among cisgender men and TGW who have sex with men compared to FTC/TDF, meeting the superiority criteria of long acting CAB (P = 0.0003) [105].

The second trial, HPTN-084 was designed for cisgender women at risk for HIV infection. Results from the study showed that at week 153, 4 (0.2%) participants in the CAB group versus 36 (1.85%) participants in the FTC/TDF group had acquired incident HIV-1 infection (hazard ratio 0.12 [95% CI 0.05–0.31]). Poor medication adherence was demonstrated when 98% (n = 35) participants in the FTC/TDF group had inadequate tenofovir and tenofovir diphosphate concentration at the time of HIV infection [106]. Of the 36 individuals who acquired HIV infection in the FTC/TDF group, only 1 participant had NRTI mutations with the primary resistance mutation being M184V**.** No major integrase RAMs were detected in any of the four HIV infections observed in the CAB group**.** By week 153, results from the study suggest that cisgender women in the CAB LA group had an 88% lower risk in acquiring HIV infection compared to those in the FTC/TDF group, thus, meeting superiority criteria of long acting CAB [106].

#### Safety and tolerability

In the HPTN-083 study, Grade 3 or higher laboratory abnormalities were reported in 31.9% of participants in the CAB group and 33.6% of participants in the FTC/TDF group. The most common laboratory abnormalities were increased creatine kinase, decreased creatinine clearance, increased serum creatinine, increased lipase, increased aspartate aminotransferase (AST), and increased alanine aminotransferase (ALT). Discontinuation because of a clinical adverse event occurred in 6% individuals in the CAB group and 4% individuals in the FTC/TDF group. Injection site-related adverse events leading to discontinuation occurred in 2.4% (n = 50) participants in the long-acting CAB group. Mean changes in weight gain from baseline differed between the treatment groups with a greater increase occurring in the CAB group (1.23 kg) versus the FTC/TDF group (0.37 kg) [105].

In the HPTN-084 study, discontinuation because of a clinical adverse event occurred in 1% in the CAB group and 1% in the FTC/TDF group**.** Adverse events leading to discontinuation occurred in two individuals in the CAB group due to hospitalization for fetal distress and respiratory tract infection**.** Grade 3 or higher laboratory abnormalities were reported in 17.1% of participants in the CAB group and 17.4% of participants in the FTC/TDF group. The most common laboratory abnormalities were decreased creatinine clearance, increased creatinine, increased creatine kinase, abnormal weight loss, increased ALT, and increased AST**.** Mean changes in weight gain from baseline did not differ significantly between treatment groups with an increase of 2.4 kg in the CAB group compared to 2.1 kg in the FTC/TDF group [106].

### Clinical considerations

**Fig. 2:**
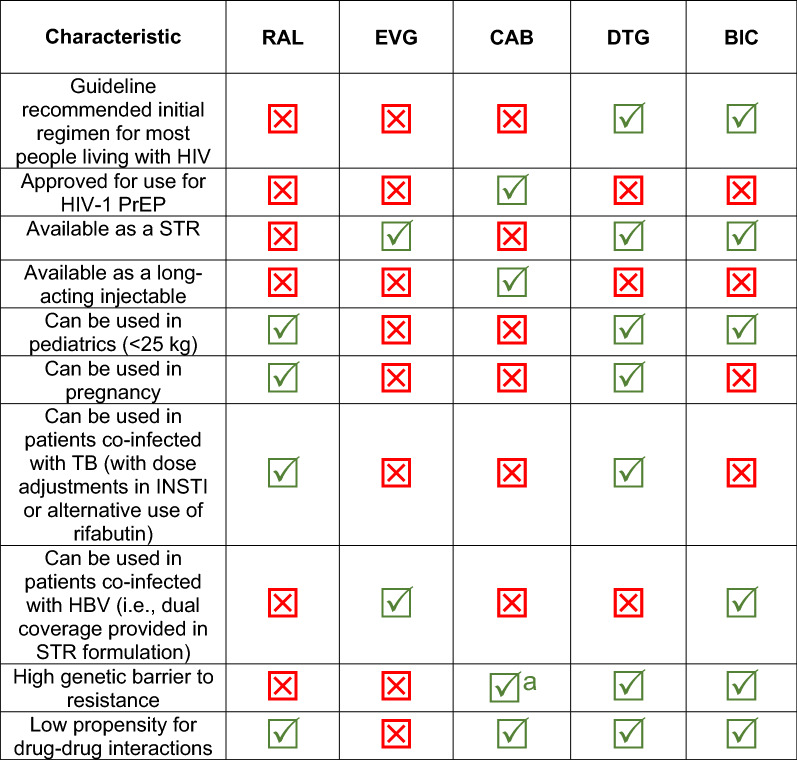
Clinical considerations for choosing between INSTIs. ^a^Cabotegravir has a higher genetic barrier to resistance than first generation INSTIs (RAL and EVG) with limited cross-resistance to these latter INSTIs; however, CAB may have a lower genetic barrier to resistance than other second generation INSTIs (DTG and BIC). *BIC* bictegravir, *CAB* cabotegravir, *DTG* dolutegravir, *EVG* elvitegravir, *HBV* hepatitis B virus, *RAL* raltegravir, *STR* single tablet regimen, *TB* tuberculosis

INSTIs used for treatment-naïve individuals living with HIV have shown excellent rates of virologic suppression at week 48 ranging from 85 to 93%, in addition to favorable tolerability versus other ARV class comparator groups. Furthermore, the efficacy of these INSTIs have been sustained with extended duration of use [67, 69, 76, 107]. Taken together, all these studies support the recommended use of INSTIs for first line treatment of individuals living with HIV. Refer to Fig. 2 for a summary of additional relevant clinical considerations for helping clinicians choose between the various INSTIs used for treatment of people living with HIV.

#### Resistance

One of the major advantages of starting treatment in individuals living with HIV with second generation INSTIs like DTG or BIC is their characteristically high genetic barrier to resistance compared to the first generation INSTIs, RAL and EVG, and other ARV classes such as NNRTIs (for example: EFV and RPV) [49, 73, 108, 109]. The high genetic barrier to development of resistance of DTG, BIC, and to a lesser extent CAB may be attributed to their slower dissociation half-lives, making them advantageous over RAL and EVG in the presence of INSTI RAMs. Reports of INSTI RAMs were detected in clinical studies involving RAL and EVG (refer to Table 4). However, negligible treatment-emergent resistance to BIC and DTG were reported in treatment-naïve individuals in clinical studies, further supporting their durable role as effective agents for treating HIV (refer to Table 6) [92, 93, 98, 110–112]. Current evidence also supports switching INSTIs from DTG to BIC with either FTC/TDF or FTC/TAF even in the presence of pre-existing NRTI resistance, further illustrating BIC’s high genetic barrier to resistance [113]. For this reason and given that transmission rates of resistance are low among INSTIs, the use of second generation INSTI containing regimens including DTG or BIC are preferred as viable options in individuals needing to initiate rapid start for treatment of HIV [2]. Additionally, although rates of treatment failure with second generation INSTIs are low, further study of resistance pathways outside of the integrase gene will lead to a greater understanding of these mechanisms and potential solutions to overcoming associated resistance.

#### Safety/adverse effects

Overall, the most common adverse effects associated with INSTIs in clinical studies were diarrhea, nausea, insomnia, fatigue, and headache. Reported adverse events leading to discontinuation tended to be lowest in clinical studies involving BIC and DTG (≤ 2%) and slightly higher for RAL and EVG groups (< 1% to 4%). Furthermore, there were significantly fewer discontinuations secondary to adverse events for those individuals treated with the INSTI EVG versus comparator arms including EFV and ATV/r, as well as with DTG versus EFV [11, 91, 93]. These findings account for one of the primary reasons why the use of INSTIs is preferred as recommended first-line choices over other drug classes such as NNRTIs and PIs.

Discontinuations of treatment due to renal adverse events mostly occurred in individuals who were receiving EVG/COBI/FTC/TDF [11, 91]. No discontinuations due to renal adverse events occurred in the EVG/COBI/FTC/TAF treatment group, thus, supporting greater tolerability of TAF versus TDF related to renal safety. Fewer renal and bone toxicities associated with TAF may be attributed to its mechanism of action. Although both TAF and TDF are oral prodrugs of TFV, TAF was developed with improved safety and efficacy compared to TDF. TAF is more stable in plasma and is metabolized inside target cells via hydrolysis by intracellular cathepsin A, which results in higher intracellular levels of the active metabolite TFV diphosphate and lower plasma levels of TFV [114, 115]. Clinical studies using TAF have shown smaller changes in creatinine clearance and less tubular proteinuria as compared to those receiving TDF with the same background ART regimen [65, 115, 116]. However, some disadvantages of TAF include worsening effects on lipids and cumulative weight gain relative to TDF [117]. Compared to TDF, which has been shown to have lipid-suppressing benefits, increases in TG and simultaneous increase in LDL and HDL cholesterol have been observed in individuals switching from TDF to TAF [118]. For these reasons, it is understandable that fixed-dose combination INSTIs are moving toward inclusion of TAF over TDF.

Small incremental changes in renal parameters were observed initially in the first few weeks after starting treatment with EVG/COBI and stabilized thereafter with extended treatment. These effects of COBI on serum creatinine and mean estimated glomerular filtration rate (eGFR, Cockcroft-Gault, mL/min) are also consistent with results from studies evaluating protease inhibitors ATV and darunavir for the treatment of HIV, further supporting the effects of COBI on tubular secretion of serum creatinine [119–123]. COBI has been associated with small increases in serum creatinine and corresponding decreases in eGFR because of its effects on tubular secretion rather than glomerular filtration of serum creatinine [10]. In vitro data have demonstrated that COBI is a weak inhibitor of human renal transporters OCT2 and MATE2-K and is a more potent inhibitor of OCTN1 and MATE1 [10]. DTG and BIC are also known to be inhibitors of renal transporters OCT2 and MATE1. Since creatinine is a substrate of MATE1, inhibition of MATE1 by COBI, DTG and BIC may provide a plausible biological explanation for the small increases in serum creatinine and slightly reduced eGFR observed in clinical studies [8, 66, 76, 97, 98]. The magnitude of these changes in eGFR is not considered to be clinically significant, since they are relatively small and did not progress with extended treatment.

Weight gain has also been observed after the initiation of INSTI-containing regimens in ART naïve individuals. BIC and DTG are associated with the largest weight gain from baseline, and generally INSTIs are associated with greater weight gain than with either NNRTIs or boosted PIs [102, 124]. The mechanism by which this weight gain occurs is not fully understood. Some studies have shown an association between ARV-related weight gain and/or body mass index changes and certain populations such as women and Black and Hispanic populations. However, further study is warranted to understand both of what predictors, if any, exist for this phenomenon, as well as the cardiovascular and metabolic implications associated with weight gain [13, 125–130].

INSTIs may exacerbate psychiatric symptoms but occur with low frequency compared to ARVs such as EFV and they rarely necessitate discontinuation [131, 132]. Although central nervous system-related adverse events such as headache, insomnia, dizziness, and depression occurred for some individuals in landmark clinical studies involving INSTIs, very few were severe enough to lead to discontinuation. It is difficult to fully grasp the impact of INSTIs on the development of neuropsychiatric events since documentation of these can vary depending on the scales used and how they are reported. However, since depression is common in the HIV population, caution and close monitoring is recommended when starting treatment with any INSTI, especially, for those who have a history of depression and are receiving treatment with an antidepressant or other psychotropic medications.

Drug-gene interactions may also play a role in increasing the risk for adverse effects in individuals treated with DTG. One observational study including 107 Japanese patients with HIV-1 infection showed that those individuals who carried reduced function variants (*6 and *28) in UGT1A1 had higher median plasma DTG concentrations and were reported to have a significantly higher cumulative incidence of neuropsychiatric adverse events (defined as dizziness, headache, insomnia, restlessness, and anxiety) than those who carried normal alleles in UGT1A1. Although the severity of the neuropsychiatric adverse events was grade 1 or 2, six subjects discontinued DTG because of these adverse events. The impact of reduced function variants in UGT1A1 (i.e., poor metabolizer status) on incidence of adverse events and treatment outcomes associated with INSTIs warrants further study. This is especially important for those population groups, where some of these variants are commonly representative (30–40%) such as in European and African American populations, and also given that DTG is recommended as a first line treatment for most individuals with HIV worldwide [133].

#### Demographics and special populations

Most of the major clinical studies involving INSTIs included predominantly white populations, males, and mean age was in the 30 s. One strength of the BIC clinical studies is the heterogeneity of the overall populations, including diverse ethnicities such as Black or African descent (30–36%) and Hispanic/Latino populations (21–26%) [97, 98]. Most of these studies also included those who had either HBV or hepatitis C virus (HCV) coinfection with comparable efficacy. Therefore, additional benefits of using EVG- or BIC-based FDCs include ability to treat HBV co-infection simultaneously. Together, this may collectively increase adherence to treating both HBV and HIV infections. It is worth noting that FDCs such as DTG/ABC/3TC, DTG/3TC and DTG plus RPV as well as the long acting CAB with RPV combination are not recommended for treatment of HBV co-infection since they do not include the standard two NRTIs recommended for treatment of HBV in patients living with HIV.

Another important demographic in these clinical studies was that the mean age was considerably young (mid-30s). Given that the HIV population is aging, additional clinical studies are warranted to determine the efficacy and safety of the use of INSTIs in elderly populations. Although this information is not fully known, one major advantage of INSTIs (especially, RAL, DTG, BIC and CAB) include fewer DDIs compared to EVG/COBI and other ARV classes such as PIs and NNRTIs. This is particularly critical in an aging HIV population with increased comorbidities and concomitant polypharmacy. Furthermore, the INSTIs RAL, DTG and BIC seem to have the least DDIs with direct acting antivirals compared to other ARV classes when treating common co-infections such as HCV [134]. With regards to another demographic variable such as gender, the INSTIs EVG and DTG demonstrated superior efficacy to their comparative groups including ATV/RTV-based regimens in treatment-naïve women living with HIV [135, 136].

In recent years, there has been increased interest in exploring potential associations between INSTIs and cardiovascular disease. An ongoing prospective, multicenter study including almost 30,000 people living with HIV examining INSTI exposure and the incidence of cardiovascular disease reported that after an average of six years of follow up, patients exposed to an INSTI experienced an increased cardiovascular risk within the first two years of INSTI exposure compared to those with no INSTI exposure. However, with continued follow-up, it was found that cardiovascular risk subsequently decreased to levels similar to those never exposed to an INSTI [137]. These findings merit further exploration into the possible relationship between INSTIs and cardiovascular disease, including attributable potential mechanisms responsible for this association and whether these risks vary by choice of INSTI.

Previously, DTG was recommended as an alternative ARV in individuals of childbearing potential who are trying to conceive or who are sexually active without using effective contraception. Reasoning for this limitation was based on a study in Botswana that showed that DTG exposure around the time of conception may be associated with an increased risk of infant neural tube defects [138]. Additional data from this same study have shown that this risk is considerably lower than original preliminary data suggested [139, 140]. Furthermore, given that these differences in rates were not significantly different to those with non-DTG containing regimens, DTG is now recommended as a preferred option for people of childbearing potential [141].

Other advantages of INSTIs include their use in other special populations such as children and acute HIV infection. One of the major advantages of RAL includes its availability in numerous various dosage formulations (especially, the chewable tablet formulation and oral suspension), which can help facilitate excellent adherence in young children living with HIV. Furthermore, RAL can also be used as early as birth. Recently in the United States there have been many approvals of additional pediatric formulations such as: (1) a dispersible tablet for oral suspension for DTG for infants as young as 4-weeks old, (2) a low FDC of BIC/FTC/TAF for children weighing at least 14 kg, and (3) a low FDC of DTG/ABC/3TC for children weighing 10–25 kg [22, 142]. It is important to note, however, that the dosing between adult and pediatric formulations cannot be interchanged on a mg-to-mg basis due to their varying pharmacokinetic profiles, specifically with DTG.

The potency of INSTIs in clinical scenarios such as acute HIV infection and rapid start lends itself well in reducing HIV viral load very quickly compared to other ARV classes, which may reduce the size and development of latent viral reservoirs, thus limiting the overall impact of HIV disease progression and transmission [143]. It is worth noting that a more rapid viral load decline such as that seen with treatment of INSTIs has been associated with a higher risk of developing immune reconstitution inflammatory syndrome (IRIS) in some studies [144, 145]. In a recent meta-analysis, it was concluded that there was no association between INSTI regimens and risk of IRIS [146]. Due to the limitations of many of the various studies conducted, further research is needed to directly assess and understand the potential association between INSTIs and the risk of IRIS.

#### Adherence

Adherence to treatment of HIV is essential for achieving positive treatment outcomes. Numerous barriers such as adverse effects and complexity of regimens may negatively influence adherence to ART. One major advantage of COBI is its ability to be co-formulated with other ARVs allowing for reduced pill burden through the development of once daily FDC regimens. Studies have shown the benefits of using once daily ART regimens including improved rates of adherence [147, 148]. Simplicity and convenience are important factors for ensuring that individuals living with HIV have sustained and adequate adherence to lifelong ART [149]. Given that infants with HIV who weigh at least 3 kg and aged ≥ 4 weeks can use the INSTI DTG or RAL, this might help improve overall adherence to ART in this population. Furthermore, for children who can swallow, DTG has a relatively small pill size, and its pediatric formulations are even smaller in diameter.

Although the long acting CAB and RPV injections are not approved for use in treatment-naïve individuals living with HIV, it provides a favorable option to replace the current ART regimen for those individuals who have a suppressed HIV viral load, have no known or suspected resistance to CAB and RPV, and have problems taking medication on a daily basis [150]. It is important to consider, however, that individuals being considered for once monthly or every two-month injections with CAB and RPV must be counseled regarding the importance of adhering to scheduled dosing visits. Using this treatment in a patient with a history of poor clinic visit attendance may put the patient at risk of viral rebound and development of resistance with missed doses. Similarly, this should be taken into consideration prior to clinicians prescribing CAB for PrEP to help reduce the risk of acquiring HIV-1 infection and development of resistance.

#### Drug-drug interactions

Although INSTIs are generally associated with fewer DDIs compared to other ARV classes such as NNRTIs and PIs, co-administration of INSTIs with supplements containing polyvalent cations may decrease the bioavailability of INSTIs leading to potential resistance and HIV disease progression [151, 152]. Some solutions that may help to manage this DDI are to take the INSTI with food to offset the extent of the DDI or to ideally space administration of the INSTI from polyvalent cations by at least 2 h before and 6 h after supplement administration [152]. Refer to Table 2 for a comprehensive summary of DDIs between INSTIs with selected co-administered drugs. Since complementary and alternative medicine (CAM) use is common among the HIV population, it is important for clinicians to frequently ask whether patients are using CAM so that this knowledge can inform whether there are any potential DDIs with ART including INSTIs [153].

One other significant DDI to highlight is co-administration of INSTIs such as RAL and DTG with antimycobacterial tuberculosis medications including rifamycins. This is particularly important since tuberculosis co-infection is more prevalent in low- and middle-income countries and transitioning to DTG-based regimens is becoming increasingly more common in these areas [154]. Greater pill burden with twice daily dosing is required for both RAL and DTG when either is introduced together with rifampin. This dosing modification helps to compensate for reduced concentrations of RAL and DTG that can occur with strong CYP and UGT1A1 inducers such as rifampin. However, both RAL and DTG can be used simultaneously with rifabutin without any necessary dose adjustments. In general, rifamycins are not recommended when using BIC, EVG and CAB (refer to Table 2).

## Conclusions

In conclusion, clinical trials have shown that INSTIs have excellent efficacy and favorable safety profiles compared to other ARV classes. Most INSTIs are available as once daily fixed-dose combinations offering numerous advantages in addition to simplified, reduced pill burden, including fewer DDIs with unboosted INSTIs (RAL, DTG, BIC and CAB) and high genetic barrier to development of resistance (especially, DTG and BIC). These characteristics are critical in an aging HIV population with increasingly prevalent comorbidities and concomitant polypharmacy. The long-acting injectable formulation of CAB may also serve well for those individuals who are virologically suppressed but have difficulty remembering to take medications on a daily basis, both as treatment for HIV-1 infection as well as PrEP for those at risk of acquiring HIV. The long-term clinical implications of cumulative weight gain associated with DTG and BIC warrant further study. Certain INSTIs may be more appropriate than others depending on the clinical situation. Therefore, careful consideration of a patient’s clinical history should be made when choosing between INSTIs, especially as they continue to be more commonly used worldwide.

## Data Availability

Not Applicable.

## Abbreviations

3TC: Lamivudine
ABC: Abacavir
ALT: Alanine aminotransferase
AST: Aspartate aminotransferase
ART: Antiretroviral therapy
ARV: Antiretroviral
ATV: Atazanavir
AUC: Area under the curve
BIC: Bictegravir
BMD: Bone mineral density
CAB: Cabotegravir
CAM: Complementary and alternative medicine
COBI: Cobicistat
CNS: Central nervous system
CYP: Cytochrome P-450
DDI: Drug-drug interaction
DNA: Deoxyribonucleic acid
DTG: Dolutegravir
DRM: Drug resistance mutation
EFV: Efavirenz
eGFR: Estimated glomerular filtration rate (Cockcroft-Gault, mL/min)
*Env*: HIV-1 envelope glycoprotein
EVG: Elvitegravir
FDA: Food and drug administration
FDC: Fixed-dose combination
FTC: Emtricitabine
HBV: Hepatitis B virus
HCV: Hepatitis C virus
HDL: High-density lipoprotein
HIV: Human immunodeficiency virus
IM: Intramuscular
INSTIs: Integrase strand transfer inhibitor
IRIS: Immune reconstitution inflammatory syndrome
LA: Long-acting
LDL: Low-density lipoprotein
MATE: Multi-drug and toxin extrusion transporter
MOI: Multiplicity of infection
NRTI: Nucleoside reverse transcriptase inhibitor
OAT: Organic anion transporter
OCT: Organic cation transporter
PDVF: Protocol-defined virologic failure
PrEP: Pre-exposure prophylaxis
RAL: Raltegravir
RAM: Resistance associated mutation
RNA: Ribonucleic acid
RPV: Rilpivirine
RTV: Ritonavir
TAF: Tenofovir alafenamide
TC: Total cholesterol
TG: Triglycerides
TGW: Transgender women
TDF: Tenofovir disoproxil fumarate
UGT: Uridine diphosphate glucuronosyltransferase

## References

[1] WHO Guidelines Approved by the Guidelines Review Committee (2021). In Consolidated guidelines on HIV prevention, testing, treatment, service delivery and monitoring: recommendations for a public health approach.

[2] Guidelines for the use of antiretroviral agents in adults and adolescents with HIV [https://clinicalinfo.hiv.gov/sites/default/files/inline-files/AdultandAdolescentGL.pdf]

[3] British HIV (2001). Association (BHIVA) guidelines for the treatment of HIV-infected adults with antiretroviral therapy. HIV Med.

[4] EACS Guidelines version 11.0. October 2021.

[5] Molina JM, Clotet B, van Lunzen J, Lazzarin A, Cavassini M, Henry K, Kulagin V, Givens N, Brennan C, de Oliveira CF (2014). Once-daily dolutegravir is superior to once-daily darunavir/ritonavir in treatment-naive HIV-1-positive individuals: 96 week results from FLAMINGO. J Int AIDS Soc.

[6] Raffi F, Jaeger H, Quiros-Roldan E, Albrecht H, Belonosova E, Gatell JM, Baril JG, Domingo P, Brennan C, Almond S, Min S (2013). Once-daily dolutegravir versus twice-daily raltegravir in antiretroviral-naive adults with HIV-1 infection (SPRING-2 study): 96 week results from a randomised, double-blind, non-inferiority trial. Lancet Infect Dis.

[7] Viani RM, Alvero C, Fenton T, Acosta EP, Hazra R, Townley E, Steimers D, Min S, Wiznia A (2015). Safety, pharmacokinetics and efficacy of dolutegravir in treatment-experienced HIV-1 infected adolescents: forty-eight-week results from IMPAACT P1093. Pediatr Infect Dis J.

[8] Walmsley S, Baumgarten A, Berenguer J, Felizarta F, Florence E, Khuong-Josses MA, Kilby JM, Lutz T, Podzamczer D, Portilla J (2015). Brief report: dolutegravir plus abacavir/lamivudine for the treatment of HIV-1 infection in antiretroviral therapy-naive patients: week 96 and week 144 results from the single randomized clinical trial. J Acquir Immune Defic Syndr.

[9] Kassahun K, McIntosh I, Cui D, Hreniuk D, Merschman S, Lasseter K, Azrolan N, Iwamoto M, Wagner JA, Wenning LA (2007). Metabolism and disposition in humans of raltegravir (MK-0518), an anti-AIDS drug targeting the human immunodeficiency virus 1 integrase enzyme. Drug Metab Dispos.

[10] German P, Liu HC, Szwarcberg J, Hepner M, Andrews J, Kearney BP, Mathias A (2012). Effect of cobicistat on glomerular filtration rate in subjects with normal and impaired renal function. J Acquir Immune Defic Syndr.

[11] DeJesus E, Rockstroh JK, Henry K, Molina JM, Gathe J, Ramanathan S, Wei X, Yale K, Szwarcberg J, White K (2012). Co-formulated elvitegravir, cobicistat, emtricitabine, and tenofovir disoproxil fumarate versus ritonavir-boosted atazanavir plus co-formulated emtricitabine and tenofovir disoproxil fumarate for initial treatment of HIV-1 infection: a randomised, double-blind, phase 3, non-inferiority trial. Lancet.

[12] Xue W, Jin X, Ning L, Wang M, Liu H, Yao X (2013). Exploring the molecular mechanism of cross-resistance to HIV-1 integrase strand transfer inhibitors by molecular dynamics simulation and residue interaction network analysis. J Chem Inf Model.

[13] Lahiri CD, Xu Y, Wang K, Alvarez JA, Sheth AN, O'Halloran J, Spence AB, Tien P, Gustafson DR, Milam J (2021). Weight and body mass index change after switching to integrase inhibitors or tenofovir alafenamide among women living with HIV. AIDS Res Hum Retroviruses.

[14] Cabenuva [package insert]. Research triangle park, NC: ViiV Healthcare; 2022. https://gskpro.com/content/dam/global/hcpportal/en_US/Prescribing_Information/Cabenuva/pdf/CABENUVA-PI-PIL-IFU2-IFU3.PDF. Accessed 25 July 2022.

[15] Biktarvy [package insert]. Foster City, CA: Gilead; 2021. https://www.gilead.com/~/media/files/pdfs/medicines/hiv/biktarvy/biktarvy_pi.pdf. Accessed 25 July 2022.

[16] Hocqueloux L, Lefeuvre S, Bois J, Valentin C, Brucato S, Alix A, Peyro-Saint-Paul L, Got L, Fournel F, Saint-Carlier E, et al. Bioavailability of solid vs. dissolved vs. crushed single-tablet of bictegravir / emtricitabine / tenofovir alafenamide in HIV negative volunteers: the SOLUBIC study. Poster presented at: 18th European AIDS Conference; October 27–30, 2021; London, United Kingdom. Abstract available at: https://www.abstractserver.com/eacsabstractarchive/. Accessed 26 Sept 2022.

[17] Roa PE, Bazzi R (2022). Crushed bictegravir/emtricitabine/tenofovir alafenamide in a human immunodeficiency virus-positive patient with pancreatic cancer. Int J STD AIDS.

[18] Rowe SM, Clary JC, Drummond M, Derrick C, Sanasi K, Bookstaver PB (2022). Increased viral load in a hospitalized patient on treatment with crushed bictegravir/emtricitabine/tenofovir alafenamide: a case report and review of the literature. Am J Health Syst Pharm.

[19] Apretude [package insert]. Research triangle park, NC: ViiV Healthcare; 2021. https://gskpro.com/content/dam/global/hcpportal/en_US/Prescribing_Information/Apretude/pdf/APRETUDE-PI-PIL-IFU.PDF. Accessed 25 July 2022.

[20] Tivicay [package insert] research triangle park NG, 2013.: Tivicay [package insert] Research triangle park, NC: GlaxoSmithKline; 2022. https://gskpro.com/content/dam/global/hcpportal/en_US/Prescribing_Information/Tivicay/pdf/TIVICAY-PI-PIL-IFU.PDF#page=1. Accessed 24 Sept 2022.

[21] Dovato [package insert]. Research triangle park, NC: ViiV healthcare; 2022. https://gskpro.com/content/dam/global/hcpportal/en_US/Prescribing_Information/Dovato/pdf/DOVATO-PI-PIL.PDF. Accessed 24 Sept 2022.

[22] Ruel TD, Acosta EP, Liu JP, Gray KP, George K, Montañez N, Popson S, Buchanan AM, Bartlett M, Dayton D (2022). Pharmacokinetics, safety, tolerability, and antiviral activity of dolutegravir dispersible tablets in infants and children with HIV-1 (IMPAACT P1093): results of an open-label, phase 1–2 trial. Lancet HIV.

[23] Roskam-Kwint M, Bollen P, Colbers A, Duisenberg-van Essenberg M, Harbers V, Burger D (2018). Crushing of dolutegravir fixed-dose combination tablets increases dolutegravir exposure. J Antimicrob Chemother.

[24] Moore SE, Huesgen E, Howe Z (2020). Sustained virologic suppression with abacavir, emtricitabine, and crushed dolutegravir and tenofovir alafenamide in a patient with HIV and eosinophilic esophagitis. Int J STD AIDS.

[25] Buscemi L (2016). Virological suppression after use of crushed tenofovir–emtricitabine and dolutegravir tablets in a patient with HIV infection. Am J Health Syst Pharm.

[26] Juluca [package insert]. Research Triangle Park, NC: ViiV Healthcare; 2022. https://gskpro.com/content/dam/global/hcpportal/en_US/Prescribing_Information/Juluca/pdf/JULUCA-PI-PIL.PDF#page=1. Accessed 24 Sept 2022.

[27] Triumeq [package insert] Research Triangle Park, NC: ViiV Healthcare; 2022. https://gskpro.com/content/dam/global/hcpportal/en_US/Prescribing_Information/Triumeq/pdf/TRIUMEQ-PI-MG-IFU.PDF#page=1. Accessed 24 Sept 2022.

[28] Genvoya [package insert] Foster City, CA: Gilead Sciences, Inc; 2022. https://www.gilead.com/~/media/files/pdfs/medicines/hiv/genvoya/genvoya_pi.pdf. Accessed 24 Sept 2022.

[29] Fulco PP, Ayala-Sims VA (2014). Sustained virological response after taking crushed elvitegravir-cobicistat-emtricitabine-tenofovir tablets. Am J Health Syst Pharm.

[30] Kaplun O, Psevdos G (2019). Sustained HIV virologic suppression with crushed combination tablets containing elvitegravir, cobicistat, emtricitabine, and tenofovir alafenamide. Am J Health Syst Pharm.

[31] Jongbloed-de Hoon M, Colbers A, Velthoven-Graafland K, Duisenberg-van Essenberg M, Kruijssen M, Abbink E, van Crevel R, Burger D (2017). Brief report: pharmacokinetics of crushed elvitegravir combination tablet given with or without enteral nutrition. J Acquir Immune Defic Syndr.

[32] Stribild [package insert] Foster City, CA: Gilead Sciences; 2021. https://www.gilead.com/~/media/files/pdfs/medicines/hiv/genvoya/genvoya_pi.pdf. Accessed 25 July 2022.

[33] Isentress [package insert] Rahway, NJ: Merck & Co, Inc; 2022. https://www.merck.com/product/usa/pi_circulars/i/isentress/isentress_pi.pdf. Accessed 25 July 2022.

[34] Akil B, Blick G, Haggins DP, Ramgopal MN, Richmond GJ, Samuel RM, Givens N, Vavro C, Song IH, Wynne B (2015). Ait-Khaled M: dolutegravir versus placebo in subjects harbouring HIV-1 with integrase inhibitor resistance associated substitutions: 48-week results from VIKING-4, a randomized study. Antiviral Therapy.

[35] Hawkins KL, Montague BT, Rowan SE, Beum R, McLees MP, Johnson S, Gardner EM (2019). Boosted darunavir and dolutegravir dual therapy among a cohort of highly treatment-experienced individuals. Antivir Ther.

[36] Davy-Mendez T, Napravnik S, Zakharova O, Wohl DA, Farel CE, Eron JJ (2019). Effectiveness of integrase strand transfer inhibitors among treatment-experienced patients in a clinical setting. AIDS.

[37] Levy ME, Griffith C, Ellenberger N, Monroe AK, Castel AD, Rakhmanina N (2020). Outcomes of integrase inhibitor-based antiretroviral therapy in a clinical cohort of treatment-experienced children, adolescents, and young adults with HIV infection. Pediatr Infect Dis J..

[38] Castagna A, Ferrara M, Galli L, Comi L, Sterrantino G, Cenderello G, Zaccarelli M, Focà E, Roncadori A, Lazzarin A, Group PS (2018). Long-term efficacy of dolutegravir in treatment-experienced subjects failing therapy with HIV-1 integrase strand inhibitor-resistant virus. J Antimicrob Chemother.

[39] Rossetti B, Fabbiani M, Di Carlo D, Incardona F, Abecasis A, Gomes P, Geretti AM, Seguin-Devaux C, Garcia F, Kaiser R (2022). Effectiveness of integrase strand transfer inhibitors in HIV-infected treatment-experienced individuals across Europe. HIV Med.

[40] Eron JJ, Clotet B, Durant J, Katlama C, Kumar P, Lazzarin A, Poizot-Martin I, Richmond G, Soriano V, Ait-Khaled M (2013). Safety and efficacy of dolutegravir in treatment-experienced subjects with raltegravir-resistant HIV type 1 infection: 24-week results of the VIKING Study. J Infect Dis.

[41] Espeseth AS, Felock P, Wolfe A, Witmer M, Grobler J, Anthony N, Egbertson M, Melamed JY, Young S, Hamill T (2000). HIV-1 integrase inhibitors that compete with the target DNA substrate define a unique strand transfer conformation for integrase. Proc Natl Acad Sci USA.

[42] Hare S, Gupta SS, Valkov E, Engelman A, Cherepanov P (2010). Retroviral intasome assembly and inhibition of DNA strand transfer. Nature.

[43] Barreca ML, Ferro S, Rao A, De Luca L, Zappalà M, Monforte AM, Debyser Z, Witvrouw M, Chimirri A (2005). Pharmacophore-based design of HIV-1 integrase strand-transfer inhibitors. J Med Chem.

[44] Smith SJ, Zhao XZ, Passos DO, Pye VE, Cherepanov P, Lyumkis D, Burke TR, Hughes SH (2021). HIV-1 integrase inhibitors with modifications that affect their potencies against drug resistant integrase mutants. ACS Infect Dis.

[45] Passos DO, Li M, Craigie R, Lyumkis D (2021). Retroviral integrase: Structure, mechanism, and inhibition. Enzymes.

[46] Hare S, Vos AM, Clayton RF, Thuring JW, Cummings MD, Cherepanov P (2010). Molecular mechanisms of retroviral integrase inhibition and the evolution of viral resistance. Proc Natl Acad Sci U S A.

[47] Saladini F, Giannini A, Boccuto A, Dragoni F, Appendino A, Albanesi E, Vicenti I, Zazzi M (2019). Comparable In vitro activities of second-generation HIV-1 integrase strand transfer inhibitors (INSTIs) on HIV-1 clinical isolates with INSTI resistance mutations. Antimicrob Agents Chemother.

[48] Cook NJ, Li W, Berta D, Badaoui M, Ballandras-Colas A, Nans A, Kotecha A, Rosta E, Engelman AN, Cherepanov P (2020). Structural basis of second-generation HIV integrase inhibitor action and viral resistance. Science.

[49] Smith SJ, Zhao XZ, Burke TR, Hughes SH (2018). Efficacies of Cabotegravir and Bictegravir against drug-resistant HIV-1 integrase mutants. Retrovirology.

[50] Hare S, Smith SJ, Métifiot M, Jaxa-Chamiec A, Pommier Y, Hughes SH, Cherepanov P (2011). Structural and functional analyses of the second-generation integrase strand transfer inhibitor dolutegravir (S/GSK1349572). Mol Pharmacol.

[51] Hightower KE, Wang R, Deanda F, Johns BA, Weaver K, Shen Y, Tomberlin GH, Carter HL, Broderick T, Sigethy S (2011). Dolutegravir (S/GSK1349572) exhibits significantly slower dissociation than raltegravir and elvitegravir from wild-type and integrase inhibitor-resistant HIV-1 integrase-DNA complexes. Antimicrob Agents Chemot.

[52] White KL, Osman N, Cuadra-Foy E, Brenner BG, Shivakumar D, Campigotto F, Tsiang M, Morganelli PA, Novikov N, Lazerwith SE (2021). Long dissociation of bictegravir from HIV-1 integrase-DNA complexes. Antimicrob Agents Chemot.

[53] DeAnda F, Hightower KE, Nolte RT, Hattori K, Yoshinaga T, Kawasuji T, Underwood MR (2013). Dolutegravir interactions with HIV-1 integrase-DNA: structural rationale for drug resistance and dissociation kinetics. PLoS ONE.

[54] Mathias AA, West S, Hui J, Kearney BP (2009). Dose-response of ritonavir on hepatic CYP3A activity and elvitegravir oral exposure. Clin Pharmacol Ther.

[55] Reese MJ, Savina PM, Generaux GT, Tracey H, Humphreys JE, Kanaoka E, Webster LO, Harmon KA, Clarke JD, Polli JW (2013). In vitro investigations into the roles of drug transporters and metabolizing enzymes in the disposition and drug interactions of dolutegravir, a HIV integrase inhibitor. Drug Metab Dispos.

[56] Zong J, Borland J, Jerva F, Wynne B, Choukour M, Song I (2014). The effect of dolutegravir on the pharmacokinetics of metformin in healthy subjects. J Int AIDS Soc.

[57] Zhang H, Custodio J, Wei X, Wang H, Wu A, Ling J, Martin H, Quirk E, Elliott C, Kearney B (2017). P176 Clinical pharmacology of the HIV integrase strand transfer inhibitor bictegravir. Sexually Transmitted Infections.

[58] Custodio J, West S, Yu A, Martin H, Graham H, Quirk E, Kearney B (2017). Lack of clinically relevant effect of bictegravir on metformin pharmacokinetics and pharmacodynamics. Open Forum Infect Dis..

[59] Bowers GD, Culp A, Reese MJ, Tabolt G, Moss L, Piscitelli S, Huynh P, Wagner D, Ford SL, Gould EP (2016). Disposition and metabolism of cabotegravir: a comparison of biotransformation and excretion between different species and routes of administration in humans. Xenobiotica.

[60] Reese MJ, Bowers GD, Humphreys JE, Gould EP, Ford SL, Webster LO, Polli JW (2016). Drug interaction profile of the HIV integrase inhibitor cabotegravir: assessment from in vitro studies and a clinical investigation with midazolam. Xenobiotica.

[61] Podany AT, Scarsi KK, Pham MM, Fletcher CV (2020). Comparative clinical pharmacokinetics and pharmacodynamics of hiv-1 integrase strand transfer inhibitors: an updated review. Clin Pharmacokinet.

[62] Quashie PK, Mesplède T, Wainberg MA (2013). Evolution of HIV integrase resistance mutations. Curr Opin Infect Dis.

[63] Cahn P, Kaplan R, Sax PE, Squires K, Molina JM, Avihingsanon A, Ratanasuwan W, Rojas E, Rassool M, Bloch M (2017). Raltegravir 1200 mg once daily versus raltegravir 400 mg twice daily, with tenofovir disoproxil fumarate and emtricitabine, for previously untreated HIV-1 infection: a randomised, double-blind, parallel-group, phase 3, non-inferiority trial. Lancet HIV.

[64] Cahn P, Sax PE, Squires K, Molina JM, Ratanasuwan W, Rassool M, Bloch M, Xu X, Zhou Y, Homony B (2018). Raltegravir 1200 mg once daily vs 400 mg twice daily, with emtricitabine and tenofovir disoproxil fumarate, for previously untreated HIV-1 infection: week 96 results from ONCEMRK, a randomized, double-blind, noninferiority trial. J Acquir Immune Defic Syndr.

[65] Sax PE, Wohl D, Yin MT, Post F, DeJesus E, Saag M, Pozniak A, Thompson M, Podzamczer D, Molina JM (2015). Tenofovir alafenamide versus tenofovir disoproxil fumarate, coformulated with elvitegravir, cobicistat, and emtricitabine, for initial treatment of HIV-1 infection: two randomised, double-blind, phase 3, non-inferiority trials. Lancet.

[66] Wohl DA, Cohen C, Gallant JE, Mills A, Sax PE, Dejesus E, Zolopa A, Liu HC, Plummer A, White KL (2014). A randomized, double-blind comparison of single-tablet regimen elvitegravir/cobicistat/emtricitabine/tenofovir DF versus single-tablet regimen efavirenz/emtricitabine/tenofovir DF for initial treatment of HIV-1 infection: analysis of week 144 results. J Acquir Immune Defic Syndr.

[67] Zolopa A, Sax PE, DeJesus E, Mills A, Cohen C, Wohl D, Gallant JE, Liu HC, Plummer A, White KL (2013). A randomized double-blind comparison of coformulated elvitegravir/cobicistat/emtricitabine/tenofovir disoproxil fumarate versus efavirenz/emtricitabine/tenofovir disoproxil fumarate for initial treatment of HIV-1 infection: analysis of week 96 results. J Acquir Immune Defic Syndr.

[68] Arribas JR, Thompson M, Sax PE, Haas B, McDonald C, Wohl DA, DeJesus E, Clarke AE, Guo S, Wang H (2017). Brief report: randomized, double-blind comparison of tenofovir alafenamide (TAF) vs tenofovir disoproxil fumarate (TDF), each coformulated with elvitegravir, cobicistat, and emtricitabine (E/C/F) for initial HIV-1 treatment: week 144 results. J Acquir Immune Defic Syndr.

[69] Clumeck N, Molina JM, Henry K, Gathe J, Rockstroh JK, DeJesus E, Wei X, White K, Fordyce MW, Rhee MS (2014). A randomized, double-blind comparison of single-tablet regimen elvitegravir/cobicistat/emtricitabine/tenofovir DF vs ritonavir-boosted atazanavir plus emtricitabine/tenofovir DF for initial treatment of HIV-1 infection: analysis of week 144 results. J Acquir Immune Defic Syndr.

[70] Hurt CB, Sebastian J, Hicks CB, Eron JJ (2014). Resistance to HIV integrase strand transfer inhibitors among clinical specimens in the United States, 2009–2012. Clin Infect Dis.

[71] Liu TF, Shafer RW (2006). Web resources for HIV type 1 genotypic-resistance test interpretation. Clin Infect Dis.

[72] Quashie PK, Mesplède T, Han YS, Oliveira M, Singhroy DN, Fujiwara T, Underwood MR, Wainberg MA (2012). Characterization of the R263K mutation in HIV-1 integrase that confers low-level resistance to the second-generation integrase strand transfer inhibitor dolutegravir. J Virol.

[73] Tsiang M, Jones GS, Goldsmith J, Mulato A, Hansen D, Kan E, Tsai L, Bam RA, Stepan G, Stray KM (2016). Antiviral activity of bictegravir (GS-9883), a novel potent HIV-1 integrase strand transfer inhibitor with an improved resistance profile. Antimicrob Agents Chemother.

[74] Rhee SY, Gonzales MJ, Kantor R, Betts BJ, Ravela J, Shafer RW (2003). Human immunodeficiency virus reverse transcriptase and protease sequence database. Nucleic Acids Res.

[75] Passos DO, Li M, Jóźwik IK, Zhao XZ, Santos-Martins D, Yang R, Smith SJ, Jeon Y, Forli S, Hughes SH (2020). Structural basis for strand-transfer inhibitor binding to HIV intasomes. Science.

[76] Raffi F, Jaeger H, Quiros-Roldan E, Albrecht H, Belonosova E, Gatell JM, Baril JG, Domingo P, Brennan C, Almond S (2013). Once-daily dolutegravir versus twice-daily raltegravir in antiretroviral-naive adults with HIV-1 infection (SPRING-2 study): 96 week results from a randomised, double-blind, non-inferiority trial. Lancet Infect Dis.

[77] Cahn P, Pozniak AL, Mingrone H, Shuldyakov A, Brites C, Andrade-Villanueva JF, Richmond G, Buendia CB, Fourie J, Ramgopal M (2013). Dolutegravir versus raltegravir in antiretroviral-experienced, integrase-inhibitor-naive adults with HIV: week 48 results from the randomised, double-blind, non-inferiority SAILING study. Lancet.

[78] Fourati S, Charpentier C, Amiel C, Morand-Joubert L, Reigadas S, Trabaud M-A, Delaugerre C, Nicot F, Rodallec A, Maillard A (2015). Cross-resistance to elvitegravir and dolutegravir in 502 patients failing on raltegravir: a French national study of raltegravir-experienced HIV-1-infected patients. J Antimicrob Chemother.

[79] Malet I, Subra F, Charpentier C, Collin G, Descamps D, Calvez V, Marcelin AG, Delelis O (2017). Mutations Located outside the integrase gene can confer resistance to HIV-1 integrase strand transfer inhibitors. Bio.

[80] Wijting IEA, Lungu C, Rijnders BJA, van der Ende ME, Pham HT, Mesplede T, Pas SD, Voermans JJC, Schuurman R, van de Vijver D (2018). HIV-1 resistance dynamics in patients with virologic failure to dolutegravir maintenance monotherapy. J Infect Dis.

[81] Wei Y, Sluis-Cremer N (2021). Mutations in the HIV-1 3’-polypurine tract and integrase strand transfer inhibitor resistance. Antim Age Chemot.

[82] Richetta C, Subra F, Malet I, Leh H, Charpentier C, Corona A, Collin G, Descamps D, Deprez E, Parissi V (2022). Mutations in the 3'-PPT Lead to HIV-1 Replication without Integration. J Virol.

[83] Wu Y, Marsh JW (2001). Selective transcription and modulation of resting T cell activity by preintegrated HIV DNA. Science.

[84] Cara A, Cereseto A, Lori F, Reitz MS (1996). HIV-1 protein expression from synthetic circles of DNA mimicking the extrachromosomal forms of viral DNA. J Biol Chem.

[85] Van Duyne R, Kuo LS, Pham P, Fujii K, Freed EO (2019). Mutations in the HIV-1 envelope glycoprotein can broadly rescue blocks at multiple steps in the virus replication cycle. Proc Natl Acad Sci U S A.

[86] Wilen CB, Tilton JC, Doms RW (2012). HIV cell binding and entry. Cold Spring Harb Perspect Med.

[87] Sattentau QJ (2010). Cell-to-cell spread of retroviruses. Viruses.

[88] Sigal A, Kim JT, Balazs AB, Dekel E, Mayo A, Milo R, Baltimore D (2011). Cell-to-cell spread of HIV permits ongoing replication despite antiretroviral therapy. Nature.

[89] Hikichi Y, Van Duyne R, Pham P, Groebner JL, Wiegand A, Mellors JW, Kearney MF, Freed EO (2021). Mechanistic analysis of the broad antiretroviral resistance conferred by HIV-1 envelope glycoprotein mutations. mBio.

[90] Lennox JL, DeJesus E, Lazzarin A, Pollard RB, Madruga JV, Berger DS, Zhao J, Xu X, Williams-Diaz A, Rodgers AJ (2009). Safety and efficacy of raltegravir-based versus efavirenz-based combination therapy in treatment-naive patients with HIV-1 infection: a multicentre, double-blind randomised controlled trial. Lancet.

[91] Sax PE, DeJesus E, Mills A, Zolopa A, Cohen C, Wohl D, Gallant JE, Liu HC, Zhong L, Yale K (2012). Co-formulated elvitegravir, cobicistat, emtricitabine, and tenofovir versus co-formulated efavirenz, emtricitabine, and tenofovir for initial treatment of HIV-1 infection: a randomised, double-blind, phase 3 trial, analysis of results after 48 weeks. Lancet.

[92] Raffi F, Rachlis A, Stellbrink HJ, Hardy WD, Torti C, Orkin C, Bloch M, Podzamczer D, Pokrovsky V, Pulido F (2013). Once-daily dolutegravir versus raltegravir in antiretroviral-naive adults with HIV-1 infection: 48 week results from the randomised, double-blind, non-inferiority SPRING-2 study. Lancet.

[93] Walmsley SL, Antela A, Clumeck N, Duiculescu D, Eberhard A, Gutiérrez F, Hocqueloux L, Maggiolo F, Sandkovsky U, Granier C (2013). Dolutegravir plus abacavir-lamivudine for the treatment of HIV-1 infection. N Engl J Med.

[94] Walmsley S, Baumgarten A, Berenguer J, Felizarta F, Florence E, Khuong-Josses MA, Kilby JM, Lutz T, Podzamczer D, Portilla J (2015). Dolutegravir plus abacavir/lamivudine for the treatment of HIV-1 infection in antiretroviral therapy-naive patients: Week 96 and week 144 results from the SINGLE randomized clinical trial. J Acquir Immune Defic Syndr.

[95] Cahn P, Madero JS, Arribas JR, Antinori A, Ortiz R, Clarke AE, Hung CC, Rockstroh JK, Girard PM, Sievers J (2020). Durable efficacy of dolutegravir plus lamivudine in antiretroviral treatment-naive adults with HIV-1 infection: 96-week results from the GEMINI-1 and GEMINI-2 randomized clinical trials. J Acquir Immune Defic Syndr.

[96] Cahn P, Madero JS, Arribas JR, Antinori A, Ortiz R, Clarke AE, Hung C-C, Rockstroh JK, Girard P-M, Sievers J (2019). Dolutegravir plus lamivudine versus dolutegravir plus tenofovir disoproxil fumarate and emtricitabine in antiretroviral-naive adults with HIV-1 infection (GEMINI-1 and GEMINI-2): week 48 results from two multicentre, double-blind, randomised, non-inferiority, phase 3 trials. The Lancet.

[97] Gallant J, Lazzarin A, Mills A, Orkin C, Podzamczer D, Tebas P, Girard PM, Brar I, Daar ES, Wohl D (2017). Bictegravir, emtricitabine, and tenofovir alafenamide versus dolutegravir, abacavir, and lamivudine for initial treatment of HIV-1 infection (GS-US-380-1489): a double-blind, multicentre, phase 3, randomised controlled non-inferiority trial. Lancet.

[98] Sax PE, Pozniak A, Montes ML, Koenig E, DeJesus E, Stellbrink HJ, Antinori A, Workowski K, Slim J, Reynes J (2017). Coformulated bictegravir, emtricitabine, and tenofovir alafenamide versus dolutegravir with emtricitabine and tenofovir alafenamide, for initial treatment of HIV-1 infection (GS-US-380-1490): a randomised, double-blind, multicentre, phase 3, non-inferiority trial. Lancet.

[99] Orkin C, Arasteh K, Górgolas Hernández-Mora M, Pokrovsky V, Overton ET, Girard PM, Oka S, Walmsley S, Bettacchi C, Brinson C (2020). Long-acting cabotegravir and rilpivirine after oral induction for HIV-1 infection. N Engl J Med.

[100] Swindells S, Andrade-Villanueva JF, Richmond GJ, Rizzardini G, Baumgarten A, Masiá M, Latiff G, Pokrovsky V, Bredeek F, Smith G (2020). Long-acting cabotegravir and rilpivirine for maintenance of HIV-1 suppression. N Engl J Med.

[101] Santos JR, Saumoy M, Curran A, Bravo I, Llibre JM, Navarro J, Estany C, Podzamczer D, Ribera E, Negredo E (2015). The lipid-lowering effect of tenofovir/emtricitabine: a randomized, crossover, double-blind, placebo-controlled trial. Clin Infect Dis.

[102] Orkin C, DeJesus E, Sax PE, Arribas JR, Gupta SK, Martorell C, Stephens JL, Stellbrink H-J, Wohl D, Maggiolo F (2020). Fixed-dose combination bictegravir, emtricitabine, and tenofovir alafenamide versus dolutegravir-containing regimens for initial treatment of HIV-1 infection: week 144 results from two randomised, double-blind, multicentre, phase 3, non-inferiority trials. The Lancet HIV.

[103] Orkin C, Oka S, Philibert P, Brinson C, Bassa A, Gusev D, Degen O, Garcia JG, Morell EB, Tan DHS (2021). Long-acting cabotegravir plus rilpivirine for treatment in adults with HIV-1 infection: 96-week results of the randomised, open-label, phase 3 FLAIR study. Lancet HIV.

[104] Swindells S, Lutz T, Van Zyl L, Porteiro N, Stoll M, Mitha E, Shon A, Benn P, Huang JO, Harrington CM (2022). Week 96 extension results of a Phase 3 study evaluating long-acting cabotegravir with rilpivirine for HIV-1 treatment. AIDS.

[105] Landovitz RJ, Donnell D, Clement ME, Hanscom B, Cottle L, Coelho L, Cabello R, Chariyalertsak S, Dunne EF, Frank I (2021). Cabotegravir for HIV prevention in cisgender men and transgender women. N Engl J Med.

[106] Delany-Moretlwe S, Hughes JP, Bock P, Ouma SG, Hunidzarira P, Kalonji D, Kayange N, Makhema J, Mandima P, Mathew C (2022). Cabotegravir for the prevention of HIV-1 in women: results from HPTN 084, a phase 3, randomised clinical trial. Lancet.

[107] Rockstroh JK, DeJesus E, Lennox JL, Yazdanpanah Y, Saag MS, Wan H, Rodgers AJ, Walker ML, Miller M, DiNubile MJ (2013). Durable efficacy and safety of raltegravir versus efavirenz when combined with tenofovir/emtricitabine in treatment-naive HIV-1-infected patients: final 5-year results from STARTMRK. J Acquir Immune Defic Syndr.

[108] Anstett K, Brenner B, Mesplede T, Wainberg MA (2017). HIV drug resistance against strand transfer integrase inhibitors. Retrovirology.

[109] Psichogiou M, Poulakou G, Basoulis D, Paraskevis D, Markogiannakis A, Daikos GL (2017). Recent advances in antiretroviral agents: potent integrase inhibitors. Curr Pharm Des.

[110] Gallant JE, Thompson M, DeJesus E, Voskuhl GW, Wei X, Zhang H, White K, Cheng A, Quirk E, Martin H (2017). Antiviral activity, safety, and pharmacokinetics of bictegravir as 10-day monotherapy in HIV-1-infected adults. J Acquir Immune Defic Syndr.

[111] Llibre JM, Hung CC, Brinson C, Castelli F, Girard PM, Kahl LP, Blair EA, Angelis K, Wynne B, Vandermeulen K (2018). Efficacy, safety, and tolerability of dolutegravir-rilpivirine for the maintenance of virological suppression in adults with HIV-1: phase 3, randomised, non-inferiority SWORD-1 and SWORD-2 studies. Lancet.

[112] Parienti JJ, Fournier AL, Cotte L, Schneider MP, Etienne M, Unal G, Perré P, Dutheil JJ, Morilland-Lecoq E, Chaillot F (2021). Forgiveness of dolutegravir-based triple therapy compared with older antiretroviral regimens: a prospective multicenter cohort of adherence patterns and HIV-RNA replication. Open Forum Infect Dis.

[113] Sax PE, Rockstroh JK, Luetkemeyer AF, Yazdanpanah Y, Ward D, Trottier B, Rieger A, Liu H, Acosta R, Collins SE (2021). Switching to bictegravir, emtricitabine, and tenofovir alafenamide in virologically suppressed adults with human immunodeficiency virus. Clin Infect Dis.

[114] Bam RA, Yant SR, Cihlar T (2014). Tenofovir alafenamide is not a substrate for renal organic anion transporters (OATs) and does not exhibit OAT-dependent cytotoxicity. Antivir Ther.

[115] Sax PE, Zolopa A, Brar I, Elion R, Ortiz R, Post F, Wang H, Callebaut C, Martin H, Fordyce MW, McCallister S (2014). Tenofovir alafenamide vs. tenofovir disoproxil fumarate in single tablet regimens for initial HIV-1 therapy: a randomized phase 2 study. J Acquir Immune Defic Syndr.

[116] Mills A, Crofoot G, McDonald C, Shalit P, Flamm JA, Gathe J, Scribner A, Shamblaw D, Saag M, Cao H (2015). Tenofovir alafenamide versus tenofovir disoproxil fumarate in the first protease inhibitor-based single-tablet regimen for initial HIV-1 therapy: A randomized phase 2 study. J Acquir Immune Defic Syndr.

[117] Venter WDF, Moorhouse M, Sokhela S, Fairlie L, Mashabane N, Masenya M, Serenata C, Akpomiemie G, Qavi A, Chandiwana N (2019). Dolutegravir plus Two Different Prodrugs of Tenofovir to Treat HIV. N Engl J Med.

[118] Kauppinen KJ, Kivelä P, Sutinen J (2019). Switching from tenofovir disoproxil fumarate to tenofovir alafenamide significantly worsens the lipid profile in a real-world setting. AIDS Patient Care STDS.

[119] Elion R, Cohen C, Gathe J, Shalit P, Hawkins T, Liu HC, Mathias AA, Chuck SL, Kearney BP, Warren DR (2011). Team G-U-S: Phase 2 study of cobicistat versus ritonavir each with once-daily atazanavir and fixed-dose emtricitabine/tenofovir df in the initial treatment of HIV infection. AIDS.

[120] Gallant JE, Koenig E, Andrade-Villanueva J, Chetchotisakd P, DeJesus E, Antunes F, Arasteh K, Moyle G, Rizzardini G, Fehr J (2013). Cobicistat versus ritonavir as a pharmacoenhancer of atazanavir plus emtricitabine/tenofovir disoproxil fumarate in treatment-naive HIV type 1-infected patients: week 48 results. J Infect Dis.

[121] Gallant JE, Koenig E, Andrade-Villanueva JF, Chetchotisakd P, DeJesus E, Antunes F, Arasteh K, Rizzardini G, Fehr J, Liu HC (2015). Brief report: cobicistat compared with ritonavir as a pharmacoenhancer for atazanavir in combination with emtricitabine/tenofovir disoproxil fumarate: week 144 results. J Acquir Immune Defic Syndr.

[122] Tashima K, Crofoot G, Tomaka FL, Kakuda TN, Brochot A, Van de Casteele T, Opsomer M, Garner W, Margot N, Custodio JM (2014). Cobicistat-boosted darunavir in HIV-1-infected adults: week 48 results of a phase IIIb, open-label single-arm trial. AIDS Res Ther.

[123] Tashima K, Crofoot G, Tomaka FL, Kakuda TN, Brochot A, Vanveggel S, Opsomer M, Garner W, Margot N, Custodio JM (2014). Phase IIIb, open-label single-arm trial of darunavir/cobicistat (DRV/COBI): Week 48 subgroup analysis of HIV-1-infected treatment-nave adults. J Int AIDS Soc.

[124] Bourgi K, Jenkins CA, Rebeiro PF, Palella F, Moore RD, Altoff KN, Gill J, Rabkin CS, Gange SJ, Horberg MA (2020). Weight gain among treatment-naïve persons with HIV starting integrase inhibitors compared to non-nucleoside reverse transcriptase inhibitors or protease inhibitors in a large observational cohort in the United States and Canada. J Int AIDS Soc.

[125] Sax PE, Erlandson KM, Lake JE, McComsey GA, Orkin C, Esser S, Brown TT, Rockstroh JK, Wei X, Carter CC (2020). Weight gain following initiation of antiretroviral therapy: risk factors in randomized comparative clinical trials. Clin Infect Dis.

[126] Ursenbach A, Max V, Maurel M, Bani-Sadr F, Gagneux-Brunon A, Garraffo R, Ravaux I, Robineau O, Makinson A, Rey D (2020). Incidence of diabetes in HIV-infected patients treated with first-line integrase strand transfer inhibitors: a French multicentre retrospective study. J Antimicrob Chemother.

[127] Erlandson KM, Wu K, Lake JE, Samuels DC, Bares SH, Tassiopoulos K, Koethe JR, Brown TT, Leonard M, Benson CA (2021). Mitochondrial DNA haplogroups and weight gain following switch to integrase strand transfer inhibitor-based antiretroviral therapy. AIDS.

[128] Kileel EM, Lo J, Malvestutto C, Fitch KV, Zanni MV, Fichtenbaum CJ, Overton ET, Okeke NL, Kumar P, Joao E (2021). Assessment of obesity and cardiometabolic status by integrase inhibitor Use in REPRIEVE: a propensity-weighted analysis of a multinational primary cardiovascular prevention cohort of people with human immunodeficiency virus. Open Forum Infect Dis.

[129] Chen YW, Anderson D, Pericone CD, Donga P (2022). Real-world assessment of weight change in african american females and hispanics with HIV-1 after initiating integrase strand-transfer inhibitors or protease inhibitors. J Health Econ Outcomes Res.

[130] Bedimo R, Adams-Huet B, Taylor B, Lake J, Luque A (2018). 538. Integrase inhibitor-based HAART Is associated with greater BMI gains in blacks, hispanics, and women. Open Forum Infect Dis.

[131] Fettiplace A, Stainsby C, Winston A, Givens N, Puccini S, Vannappagari V, Hsu R, Fusco J, Quercia R, Aboud M, Curtis L (2017). Psychiatric symptoms in patients receiving dolutegravir. J Acquir Immune Defic Syndr.

[132] Harris M, Larsen G, Montaner JS (2008). Exacerbation of depression associated with starting raltegravir: a report of four cases. AIDS.

[133] Yagura H, Watanabe D, Kushida H, Tomishima K, Togami H, Hirano A, Takahashi M, Hirota K, Ikuma M, Kasai D (2017). Impact of UGT1A1 gene polymorphisms on plasma dolutegravir trough concentrations and neuropsychiatric adverse events in Japanese individuals infected with HIV-1. BMC Infect Dis.

[134] Recommendations for testing, managing, and treating hepatitis C [http://www.hcvguidelines.org.]

[135] Squires K, Kityo C, Hodder S, Johnson M, Voronin E, Hagins D, Avihingsanon A, Koenig E, Jiang S, White K (2016). Integrase inhibitor versus protease inhibitor based regimen for HIV-1 infected women (WAVES): a randomised, controlled, double-blind, phase 3 study. Lancet HIV.

[136] Orrell C, Hagins DP, Belonosova E, Porteiro N, Walmsley S, Falcó V, Man CY, Aylott A, Buchanan AM, Wynne B (2017). Fixed-dose combination dolutegravir, abacavir, and lamivudine versus ritonavir-boosted atazanavir plus tenofovir disoproxil fumarate and emtricitabine in previously untreated women with HIV-1 infection (ARIA): week 48 results from a randomised, open-label, non-inferiority, phase 3b study. Lancet HIV.

[137] Neesgaard B, Greenberg L, Miró JM, Grabmeier-Pfistershammer K, Wandeler G, Smith C, De Wit S, Wit F, Pelchen-Matthews A, Mussini C (2022). Associations between integrase strand-transfer inhibitors and cardiovascular disease in people living with HIV: a multicentre prospective study from the RESPOND cohort consortium. Lancet HIV.

[138] Zash R, Makhema J, Shapiro RL (2018). Neural-tube defects with dolutegravir treatment from the time of conception. N Engl J Med.

[139] Zash R, Holmes L, Diseko M, Jacobson DL, Brummel S, Mayondi G, Isaacson A, Davey S, Mabuta J, Mmalane M (2019). Neural-tube defects and antiretroviral treatment regimens in botswana. N Engl J Med.

[140] Raesima MM, Ogbuabo CM, Thomas V, Forhan SE, Gokatweng G, Dintwa E, Petlo C, Motswere-Chirwa C, Rabold EM, Tinker SC (2019). Dolutegravir use at conception—additional surveillance data from botswana. N Engl J Med.

[141] Panel on treatment of HIV during pregnancy and prevention of perinatal transmission. Recommendations for the use of antiretroviral drugs during pregnancy and interventions to reduce perinatal HIV transmission in the United States. Available at https://clinicalinfo.hiv.gov/en/guidelines/perinatal. Accessed Sept 2022.

[142] Natakunda E, Rodriguez CA, McGrath EJ, Hellstrom E, Liberty A, Chokephaibulkit K, Kosalaraksa P, Wong P, Hindman J, German P. B/F/TAF in virologically suppressed adolescents and children: two-year outcomes in 6 to <18 year olds and six-month outcomes in toddlers. In 13th international workshop on HIV pediatrics: 11th IAS conference on HIV science; July; virtual meeting. 2021.

[143] Llibre JM, Martinez-Picado J (2008). Potential of integrase inhibitors to deplete HIV reservoirs or prevent their replenishment. Enferm Infecc Microbiol Clin.

[144] Gaillet A, Calin R, Flandre P, Tubiana R, Valantin MA, Caumes E, Katlama C, Pourcher V (2021). Increased risk of IRIS-associated tuberculosis in HIV-infected patients receiving integrase inhibitors. Infect Dis Now.

[145] Psichogiou M, Basoulis D, Tsikala-Vafea M, Vlachos S, Kapelios CJ, Daikos GL (2017). Integrase strand transfer inhibitors and the emergence of immune reconstitution inflammatory syndrome (IRIS). Curr HIV Res.

[146] Zhao Y, Hohlfeld A, Namale P, Meintjes G, Maartens G, Engel ME (2022). Risk of immune reconstitution inflammatory syndrome with integrase inhibitors versus other classes of antiretrovirals: a systematic review and meta-analysis of randomized trials. J Acquir Immune Defic Syndr.

[147] Molina JM (2008). Efficacy and safety of once-daily regimens in the treatment of HIV infection. Drugs.

[148] Sweet D, Song J, Zhong Y, Signorovitch J (2014). Real-world medication persistence with single versus multiple tablet regimens for HIV-1 treatment. J Int AIDS Soc.

[149] Crutchley RD, Guduru RC, Cheng AM (2016). Evaluating the role of atazanavir/cobicistat and darunavir/cobicistat fixed-dose combinations for the treatment of HIV-1 infection. HIV AIDS (Auckl).

[150] Cabenuva [package insert]. Research Triangle Park, NC: ViiV Healthcare. 2021.

[151] Griessinger JA, Hauptstein S, Laffleur F, Netsomboon K, Bernkop-Schnurch A (2016). Evaluation of the impact of multivalent metal ions on the permeation behavior of Dolutegravir sodium. Drug Dev Ind Pharm.

[152] Song I, Borland J, Arya N, Wynne B, Piscitelli S (2015). Pharmacokinetics of dolutegravir when administered with mineral supplements in healthy adult subjects. J Clin Pharmacol.

[153] Bahall M (2017). Prevalence, patterns, and perceived value of complementary and alternative medicine among HIV patients: a descriptive study. BMC Complement Altern Med.

[154] Vitoria M, Hill A, Ford N, Doherty M, Clayden P, Venter F, Ripin D, Flexner C, Domanico PL (2018). The transition to dolutegravir and other new antiretrovirals in low-income and middle-income countries: what are the issues?. AIDS.

